# A flavin-dependent monooxygenase catalyzes the initial step in cyanogenic glycoside synthesis in ferns

**DOI:** 10.1038/s42003-020-01224-5

**Published:** 2020-09-11

**Authors:** Sara Thodberg, Mette Sørensen, Matteo Bellucci, Christoph Crocoll, Amalie Kofoed Bendtsen, David Ralph Nelson, Mohammed Saddik Motawia, Birger Lindberg Møller, Elizabeth Heather Jakobsen Neilson

**Affiliations:** 1 Plant Biochemistry Laboratory, Department of Plant and Environmental Sciences, University of Copenhagen, Thorvaldsensvej 40, 1871 Frederiksberg C, Copenhagen Denmark; 2 VILLUM Center for Plant Plasticity, University of Copenhagen, Thorvaldsensvej 40, 1871 Frederiksberg C, Copenhagen Denmark; 3 Novo Nordisk Foundation Center for Protein Research, Protein Production and Characterization Platform, University of Copenhagen, Blegdamsvej 3, 2200 Copenhagen N, Denmark; 4 Section for Plant Molecular Biology, Department of Plant and Environmental Sciences, University of Copenhagen, Thorvaldsensvej 40, 1871 Frederiksberg C, Copenhagen Denmark; 5 Department of Microbiology, Immunology and Biochemistry, University of Tennessee, 858 Madison Ave. Suite G01, Memphis, TN 38163 USA; 6 Center for Synthetic Biology, University of Copenhagen, Thorvaldsensvej 40, 1871 Frederiksberg C, Copenhagen Denmark

**Keywords:** Molecular biology, Evolution, Plant sciences

## Abstract

Cyanogenic glycosides form part of a binary plant defense system that, upon catabolism, detonates a toxic hydrogen cyanide bomb. In seed plants, the initial step of cyanogenic glycoside biosynthesis—the conversion of an amino acid to the corresponding aldoxime—is catalyzed by a cytochrome P450 from the CYP79 family. An evolutionary conundrum arises, as no CYP79s have been identified in ferns, despite cyanogenic glycoside occurrence in several fern species. Here, we report that a flavin-dependent monooxygenase (fern oxime synthase; FOS1), catalyzes the first step of cyanogenic glycoside biosynthesis in two fern species (*Phlebodium aureum* and *Pteridium aquilinum*), demonstrating convergent evolution of biosynthesis across the plant kingdom. The FOS1 sequence from the two species is near identical (98%), despite diversifying 140 MYA. Recombinant FOS1 was isolated as a catalytic active dimer, and in planta, catalyzes formation of an N-hydroxylated primary amino acid; a class of metabolite not previously observed in plants.

## Abstract (web summary)

Thodberg et al. report the first step of the synthesis of cyanogenic glycosides, a plant defense mechanism, by a Flavin-dependent monooxygenase (FOS1) in ferns, revealing convergent evolution of biosynthesis across the plant kingdom. The authors isolate FOS1 as a catalytically active dimer and demonstrate that it catalyzes the formation of N-hydroxylated amino acids.

## Introduction

Plants produce a plethora of natural products (phytochemicals or specialized metabolites) enabling interactions with their biotic and abiotic environment. Cyanogenic glycosides are one such class of amino acid-derived natural products present in more than 3000 plant species, including ferns, gymnosperms, and angiosperms^1–3^. For example, the cyanogenic glycosides prunasin and amygdalin are responsible for the bitterness of wild almond (*Prunus dulcis*)^4,5^. Upon tissue disruption, cyanogenic glycosides are hydrolyzed by specific β-glucosidases resulting in detonation of a hydrogen cyanide bomb as an immediate toxic chemical response e.g. towards chewing herbivores^2^. More recently, cyanogenic glycosides have been shown to possess alternative functions as remobilizable storage molecules of reduced nitrogen, controllers of bud break and flower induction, and as quenchers of reactive oxygen species^6–9^.

**Fig. 1:**
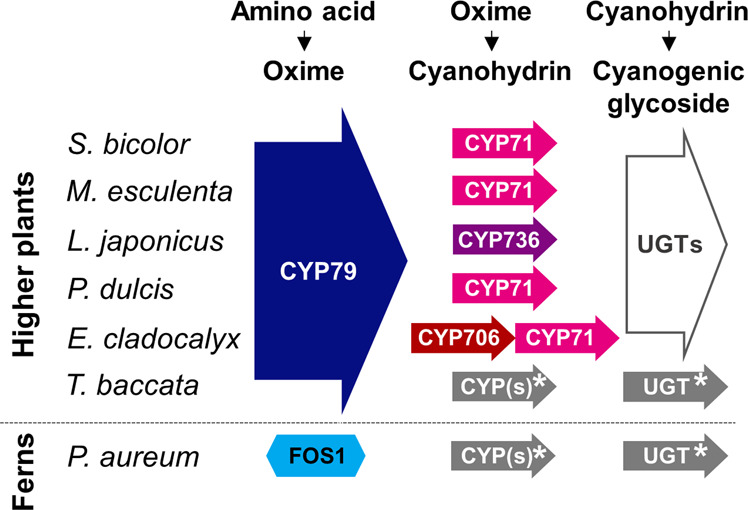
A schematic overview of the biosynthetic pathway of cyanogenic glycosides in plants. In ferns, the conversion of the parent amino acid into an oxime is catalyzed by a multifunctional FMO, whereas in all higher seed plant species analyzed, the reaction is catalyzed by a cytochrome P450 from the CYP79 family^2,4,10,12,14,61,73–79^. *Uncharacterized pathway partners.

In higher plants (gymnosperms and angiosperms), the biosynthesis of cyanogenic glycosides is catalyzed by cytochromes P450 (CYPs) and UDP-glucosyltransferases (UGTs)^2,10,11^. In all cases, initial conversion of the parent amino acid to the corresponding E-oxime is catalyzed by a functionally conserved CYP79 family enzyme. Independently evolved CYP71, CYP706, or CYP736 enzymes convert the oxime into an α-hydroxynitrile^10,12,13^ that is glycosylated by a UGT85 or UGT94 family member to produce the cyanogenic mono- or diglycosides^2,4,14^ (Fig. 1).

The CYP79-catalyzed reaction proceeds via two N-hydroxylations, a decarboxylation, and a dehydration reaction in a single catalytic site^15–17^. No other enzymes across the plant kingdom are known to catalyze the conversion of an α-amino acid into an oxime. In addition to their involvement as intermediates in cyanogenic glycoside biosynthesis, CYP79-formed oximes are important metabolites in general and specialized metabolism^18^ exemplified by indole-3-acetaldoxime, which is a shared precursor for the phytohormone auxin (indole-3-acetic acid, IAA), the phytoalexin camalexin and tryptophan-derived glucosinolates^19–21^. An evolutionary conundrum arises as no gene sequences encoding CYP79s have been found in fern transcriptomes nor genomes^22,23^ despite the occurrence of cyanogenic glycosides in ferns^1,24^. Due to their significant phylogenetic position, ferns represent an important lineage for studying the evolution of land plants. Fern research has been hampered by the scarcity of genome information^22^. In total, 11 orders of ferns are known of which four are extant: Psilotopsida, Equisetopsida, Marattiopsida, and Polypodiopsida^25^. Polypodiopsida are termed modern ferns, and ~3% of the species in this order have been reported as cyanogenic^1,24,26^.

Here, we investigate the cyanogenic glycoside biosynthetic pathway in modern ferns by a differentially expression survey of de novo assembled transcriptomes from *Phlebodium aureum* and *Pteridium aquilinum*. We report biochemical and biological evidence that ferns harbor an N-hydroxylating flavin-dependent monooxygenase that converts phenylalanine to a corresponding oxime via *N*-hydroxyphenylalanine. This demonstrates convergent evolution at the biochemical pathway level and resolves how ferns produce cyanogenic glucosides in the absence of CYP79 encoding genes.

## Results

### Metabolite-guided pathway discovery in ferns

**Fig. 2:**
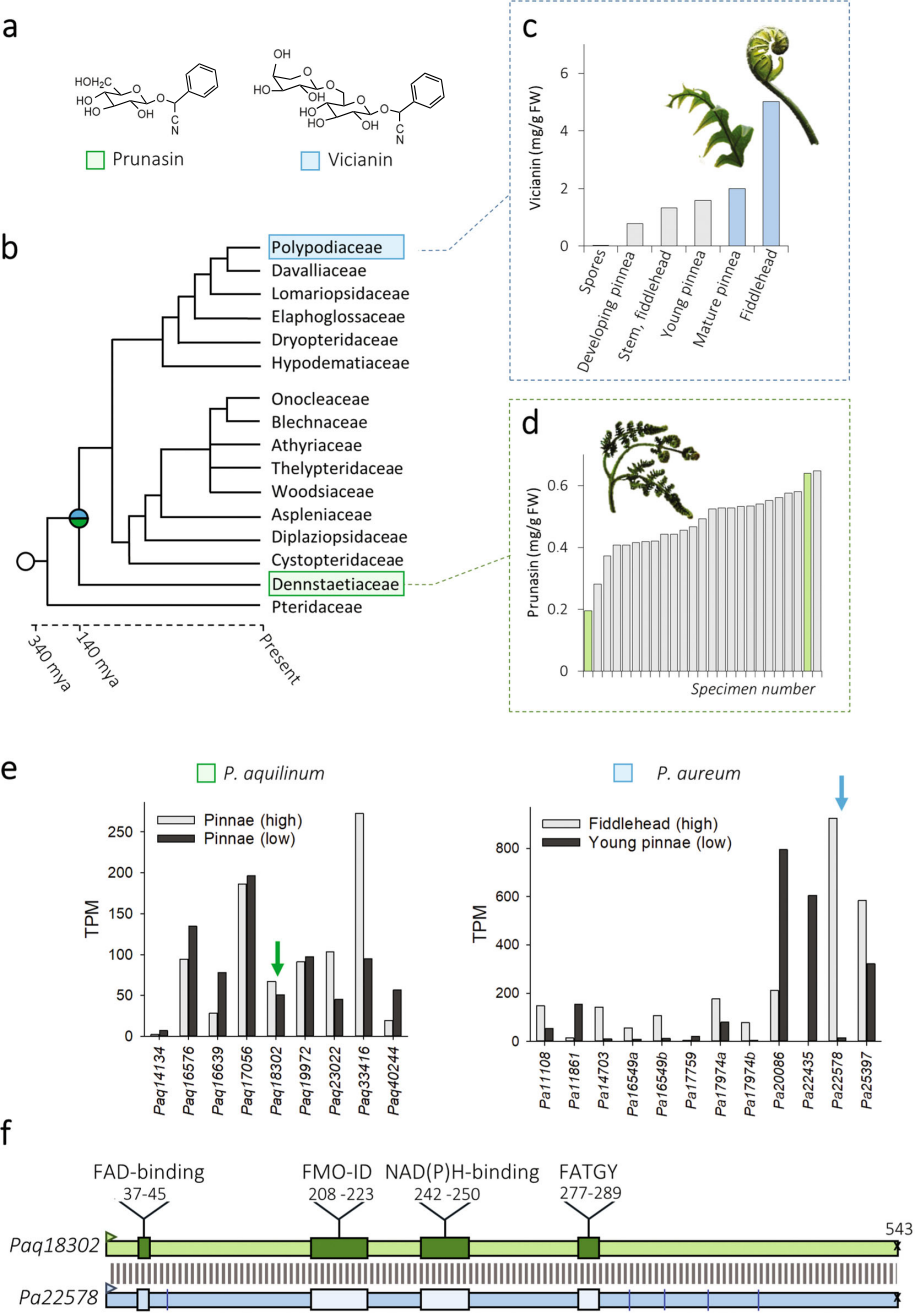
Cyanogenic glycoside content and FMO transcript abundances in tissues from the two modern fern species *Pteridium aquilinum* and *Phlebodium aureum*. **a** Two phenylalanine-derived cyanogenic glycosides have been reported from ferns: the monoglucoside prunasin (D-mandelonitrile-β-D-glucopyranoside) and the diglycoside vicianin (6-*O*-arabinopyranosylglucopyranoside). **b** Phylogenetic relationship between *Pteridium aquilinum* (Dennstaedtiaceae) and *Phlebodium aureum* (Polypodiaceae) showing that these two modern ferns species diversified 140 million years ago (tree adapted from^43^). **c** Content of vicianin across different tissue types of *P. aureum* (see also Supplementary Fig. 1), with the blue bars indicating the tissue selected for downstream transcriptomic analysis. **d** Content of prunasin in the pinnae of a population of 25 field-collected *P. aquilinum*, with the green bars indicating individuals selected for transcriptomic analysis. **e** Transcript abundance of predicted flavin monooxygenases (FMOs) in tissues of *P. aquilinum* and *P. aureum* containing high (gray bars) or low (black bars) cyanogenic glycoside levels. Arrows indicate candidate genes. TPM transcripts per million mapped reads. **f** Schematic illustration of identity and motifs in the transcriptome-deduced amino acid sequences of *Paq*18302 (*Paq*FOS1) and *Pa*22578. Differences between *Pa*22578 and the isolated *Pa*FOS1 are indicated by blue lines. The position of putative binding motifs for FAD and NADPH, the FATGY and FMO-identifier motifs conserved across plant FMOs are highlighted. Supporting alignment is shown in Supplementary Fig. 1.

The two distantly related modern ferns, *Phlebodium aureum* (Polypodiaceae) and *Pteridium aquilinum* (Dennstaedtiaceae) (Fig. 2), produce the phenylalanine-derived cyanogenic monoglucoside prunasin (D-mandelonitrile-β-D-glucopyranoside)^1,27^ and the diglycoside vicianin (6-*O*-arabinopyranosylglucopyranoside)^28^, respectively. Targeted metabolite profiling of a population of 25 field-collected *P. aquilinum* (*Paq*) identified individuals with high and low cyanogenic glycoside containing pinnae ranging from 0.2 to 0.6 mg prunasin g^−1^ fw (Fig. 2d). Two individuals were selected based on their metabolite content and the quality of RNA extracted. Similarly, analysis of different tissues within a single *Phlebodium aureum* (*Pa*) fern identified variable levels of vicianin in the different tissue types from negligible levels in the spores, to 5 mg vicianin g^−1^ fw in the emerging fiddlehead (Fig. 2c, Supplementary Fig. 1). For *Pa*, fiddlehead and young pinna were selected for transcriptome analysis (Fig. 2c). mRNA was isolated from these four tissues to obtain biosynthetic gene candidates using a comparative transcriptomic approach.

**Fig. 3:**
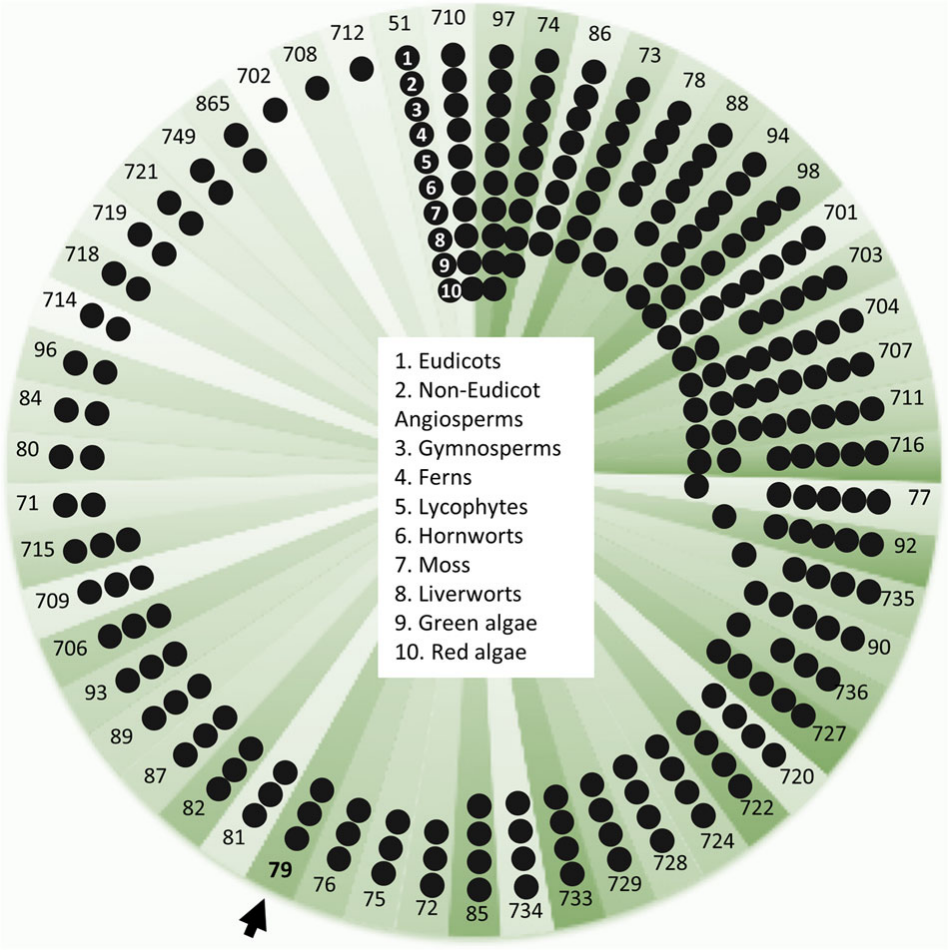
Schematic diagram illustrating the occurrence of CYP families across plant taxa, based on the known CYP families present in eudicots. The diagram is based on analysis of the OneKP database^31^, which includes transcriptomes from 74 ferns. The presence of 8930 cytochromes P450 fern sequences were predicted and sorted into families in accordance with nomenclature. The analyzed fern transcriptomes harbor at least 81 different P450 families of which 49 (60%) are novel fern-specific families. Approximately half of the CYP families present in higher plants are also found in ferns. The CYP79 family is present from gymnosperm to eudicots, but based on the >40% sequence identity, the CYP79 is absent in ferns. This also applies to the other known CYP families involved in cyanogenic glucoside biosynthesis in plants: the CYP71, CYP706, and CYP736 families.

BLAST searches of the de novo assembled transcriptomes from *Paq* and *Pa* identified 139 *CYP*s in *Paq* compared to 120 in *Pa* (Supplementary Table 1). Neither genes encoding CYP79s nor CYPs with the CYP79 signature F/H substitution in the PERF region were identified^29,30^. Additional searches of the gene sequences deposited in the OneKP database^31^ confirmed the previously reported absence of CYP79 encoding gene sequences in ferns (Fig. 3)^22,23^. In the absence of CYP candidates, we extended the search to other gene families encoding monooxygenases with focus on genes showing interesting differential expression patterns of gene homologs within and between the two fern species.

The contig *Pa*22758 encoded a flavin-dependent monooxygenase ORF of 543 aa in accordance with a predicted full-length sequence^32^. Deep-mining of the transcriptome reads revealed that *Pa* contained variants of *Pa*22758 (Supplementary Fig. 2). The transcript level of this gene was 24-fold higher (−logFDR of 4.32) in *Pa* fiddlehead compared to the young pinnae (Fig. 2e). A reciprocal BLAST search between the predicted flavin-dependent monooxygenases of *Paq* and *Pa* identified a contig *Paq*18302 in *Paq* harboring a full-length nucleotide sequence encoding a flavin-dependent monooxygenase identical to the sequence of *Pa*22758, except for a single amino acid substitution. The expression level of *Paq*18302 showed a 23% increase (−logFDR of 0.50) in the individual containing higher levels of prunasin (Fig. 2e). The deduced amino acid sequences of the *Pa* and *Paq* flavin-dependent monooxygenases revealed that they contain conserved motifs specific to Class B flavin-dependent monooxygenases (Fig. 2f, Supplementary Fig. 2)^33^.

A BLAST search in the CATH: Protein Structure Classification Database (cathdb.info, version 4.2) with the *Paq*18302 protein sequence, classified the fern sequences as belonging to the Class B, flavin-dependent monooxygenases. These enzymes are characterized by being single-component FAD-binding enzymes harboring binding sites for the hydride electron donor NAD(P)H and molecular oxygen (CATH code 3.50.50.60)^32,34,35^.

### Biochemical characterization of fern oxime synthase 1 (FOS1) in planta

**Fig. 4:**
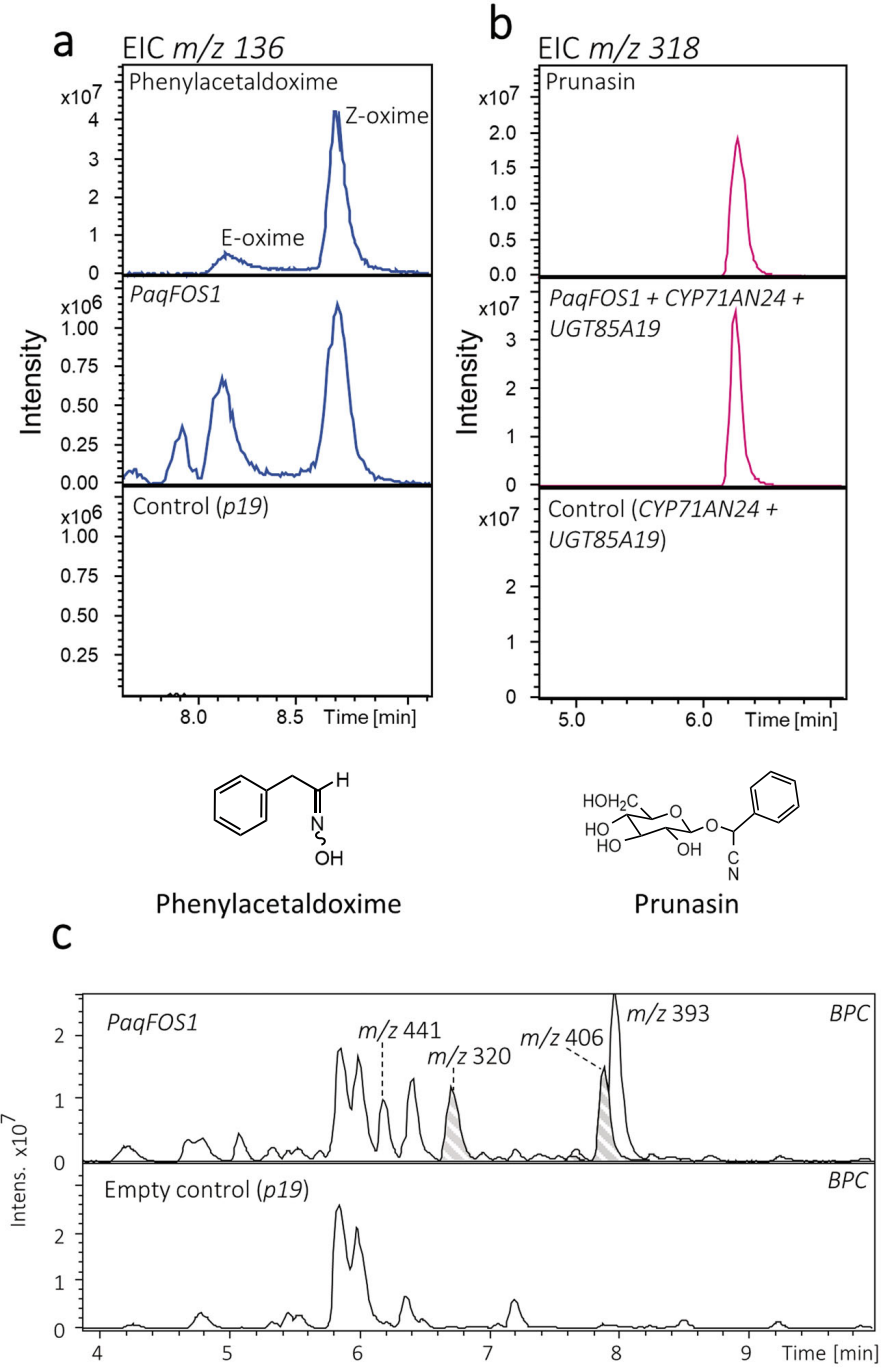
LC–MS based metabolite analyses of *Nicotiana benthamiana* leaves transiently expressing *PaqFOS1*. **a** Extracted ion chromatograms (EICs) for *m*/*z* 136 corresponding to the [M+H]^+^ adduct of authentic phenylacetaldoxime (upper panel), metabolite extracts from *N. benthamiana* leaves expressing *PaqFOS1* (middle panel) and empty vector control (lower panel). **b** *m*/*z* 318 EICs corresponding to the [M+Na]^+^ adduct of an authentic prunasin standard (upper panel), metabolite extracts from *N. benthamiana* transiently expressing *PaqFOS1* in combination with *PdCYP71AN24* and *PdUGT85A19*^4,14^ (middle panel) and the control expressing *PdCYP71AN24* and *PdUGT85A19* (lower panel). **c** Base peak chromatograms (BPCs) of the metabolite extracts from *N. benthamiana* leaves expressing *PaqFOS1* using expression of *p19* as an empty vector control show the formation of additional products: *m*/*z* 320 at 6.7 min corresponds to the [M+Na]^+^ adduct of glucosylated phenylacetaldoxime; *m*/*z* 406 at 7.6 and 7.9 min correspond to the [M+Na]^+^ adduct of a glycosylated, phenylacetaldoxime-malonic acid conjugate; and *m*/*z* 393.11 at 8.1 min correspond to the [M+Na]^+^ adduct of phenylethanol glucoside malonate ester^2,10^. For MS/MS of additional products, see Supplementary Fig. 3.

Functional characterization of the FMO enzyme *Paq*18302 (Fig. 4a) was obtained by *Agrobacterium tumefaciens*-mediated transient expression of the encoding gene in *Nicotiana benthamiana*. Leaf discs of agro-infiltrated tissue were harvested after 4 days and subjected to metabolite profiling. Expression of the *Paq*18302 FMO afforded production of two constituents of *m*/*z* 136 eluting at rt = 8.17 and 8.70 min corresponding to the [M+H]^+^ adduct of (*E*)- and (*Z*)-phenylacetaldoxime, respectively, as verified by co-elution with an authentic standard (Fig. 4a). Two additional constituents were identified as a glucoside of phenylacetaldoxime (*m*/*z* 320, [M+Na]^+^ at rt = 6.7 min) and as a phenylacetaldoxime glucoside-malonic acid conjugate (*m*/*z* 406, [M+Na]^+^ at rt = 7.9 min) based on the diagnostic fragments in the MS/MS spectra (Fig. 4c, Supplementary Fig. 3)^36–38^. Identical oxime derivatives have previously been observed in *N. benthamiana* in response to expression of CYP79 enzymes^10^. Guided by *Pa*22758, the FMO encoding sequence from *Pa* was isolated from cDNA and shown by transient expression to be functionally equivalent to the *Paq*18302 FMO (Fig. 4, Supplementary Fig. 4). We designate the orthologous FMO proteins as “FOS1”. Transient expression of *FOS1* in *N. benthamiana* did not give rise to formation of other oximes or additional products, demonstrating that FOS1 has phenylalanine as its specific amino acid substrate.

Independent functional characterization of the *FOS1* genes was obtained in *N. benthamiana* by co-expression with *CYP71AN24* and *UGT85A19*, encoding the last two enzymes in the prunasin biosynthetic pathway in almond (*Prunus dulcis*)^4,14^. This resulted in production of prunasin as demonstrated by the formation of a constituent comigrating with an authentic standard with an extracted ion chromatogram (EIC) of *m*/*z* 318 corresponding to the [M+Na]^+^ adduct of prunasin (Fig. 4b, Supplementary Fig. 4). Neither phenylacetaldoxime nor prunasin are endogenous constituents of *N. benthamiana*.

### In vitro assays revealed existence of *N*-hydroxyphenylalanine

**Fig. 5:**
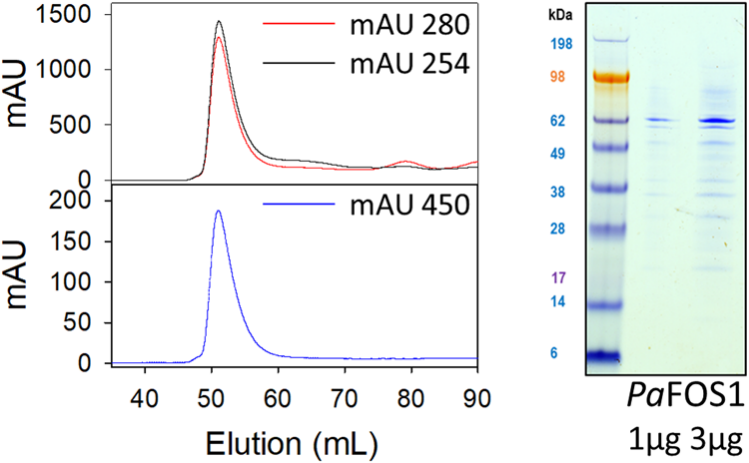
Size exclusion chromatography elution profile of *Paq*FOS1 monitored at 280, 254, and 450 nm corresponding to absorbance (mAU) of the polypeptide, NADPH, and FAD, respectively. Based on the elution volume in comparison to a set of reference proteins with known molecular masses, the *Paq*FOS1 protein eluted as a dimer with a mass of ~120 kDa. SDS-PAGE analysis of the *Paq*FOS1 containing fractions obtained by size exclusion chromatography demonstrating that *Paq*FOS1 migrated with an apparent molecular mass of 60 kDa in agreement with a calculated molecular mass of 62.5 kDa for the monomeric protein.

**Fig. 6:**
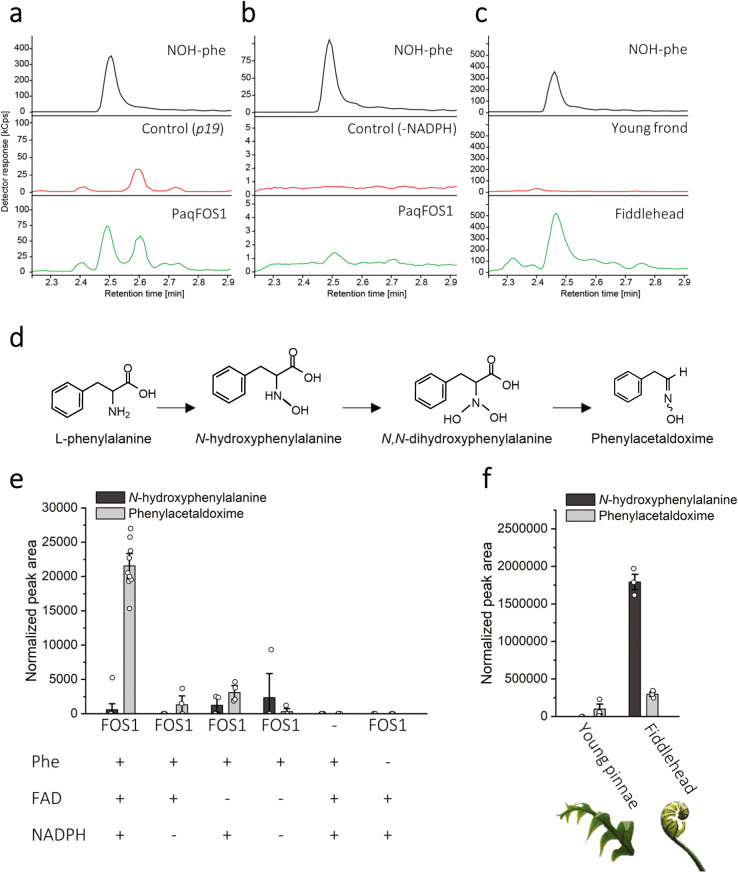
The formation of *N*-hydroxyphenylalanine (NOH-phe). LC–MS chromatograms from targeted analysis of samples compared with the authentic standard, derived from **a** transient expression of FOS1 in *N. benthamiana* showing the p19 as a negative control, **b** heterologous expression of FOS1 in *E. coli* using the absence of NADPH as a negative control, and **c** the presence of *N*-hydroxyphenylalanine in *P. aureum* tissue. The chromatographic trace represents the abundance of the fragment ion of *N*-hydroxyphenylalanine (precursor ion → fragment ion of 182.1 → 136.0; see Supplementary Table 4). **d** The hypothesized biosynthetic route from phenylalanine to phenylacetaldoxime, as catalyzed by FOS1. **e** In vitro activity assay of recombinant *Paq*FOS1 using l-phenylalanine (Phe) as a substrate, with different combinations of the necessary cofactors FAD and NADPH. Bars represent mean ± SE (*n* ≥ 3). **f** The presence of *N*-hydroxyphenylalanine in *P. aureum* fiddlehead and young pinnae metabolite extracts (*n* = 3).

Of the limited number of FMOs characterized from plants, only YUCCA6 from *A. thaliana* and *As*FMO from garlic (*Allium sativum*) have been successfully expressed and purified in enough quantity for downstream biochemical analyses^33,39,40^. Here, we expressed the *Paq*FOS1 protein with an N-terminal 6xHIS tag using *Escherichia coli* as a heterologous host and isolated *Paq*FOS1 by immobilized metal affinity chromatography followed by size exclusion chromatography (SEC) with a yield of 0.7 mg/L culture (Fig. 5). The isolated *Paq*FOS1 protein binds the cofactors FAD and NADPH as demonstrated by absorbance spectrometry, and migrates with an apparent molecular mass of 60 kDa on SDS-PAGE (Fig. 5) in agreement with a calculated molecular mass of 62.5 kDa. Upon SEC, *Paq*FOS1 eluted with a mass of 120 kDa suggesting that the native protein is a homodimer. In vitro assays of the isolated *Paq*FOS1 protein followed by LC–MS analyses confirmed that FOS1 can catalyze the conversion of phenylalanine to phenylacetaldoxime (Fig. 6). Concomitant production of *N*-hydroxyphenylalanine was also observed and verified using a chemically synthesized standard (Supplementary Fig. 8). Surprisingly, targeted LC–MS analysis of fiddleheads of *Pa* as well as of *N. benthamiana* leaves transiently expressing *FOS1* demonstrated the in vivo presence of *N*-hydroxyphenylalanine in these tissues (Fig. 6, Supplementary Fig. 5).

## Discussion

In this report, we identify and functionally characterize a flavin-dependent monooxygenase designated *FOS1* from two modern fern species, *Pa* and *Paq*. Metabolite profiling of *N. benthamiana* leaves following transient expression of *PaqFOS1* revealed the production of phenylacetaldoxime, which was also confirmed by the targeted in vitro experiments. When jointly expressed with *CYP71AN24* and *UGT85A19* from almond (*Prunus dulcis*), the entire prunasin pathway was established.

The FOS1 enzyme belongs to FMO class B, which are single-component FAD-binding enzymes that also harbor binding sites for the hydride electron donor NAD(P)H and molecular oxygen^34,41^. *Paq*FOS1 was isolated and purified as a functionally active homodimer. In vitro assays demonstrated that FOS1 is able to convert its substrate phenylalanine into two products, *N*-hydroxyphenylalanine and phenylacetaldoxime. The first product is obtained by a single N-hydroxylation reaction, the second by two consecutive N-hydroxylations followed by decarboxylation and dehydration reactions. In plants, the conversion of an amino acid to the corresponding aldoxime has only been reported as catalyzed by P450s from the CYP79 family. It is to be noted that an FMO from the actinomycete fungus *Streptomyces coelicolor* A3 can convert tryptophan and C5 prenylated tryptophan into their corresponding aldoximes^42^. Free *N*-hydroxyphenylalanine was also present in the fern tissue of *Pa*. N-hydroxylated protein amino acids have to our knowledge not previously been detected in biological tissues, and their functional roles remains unknown.

The 98% conservation of the *FOS1* encoding gene sequences of *Pa* and *Paq* is remarkable. Among the total number of 55,000 and 63,000 gene sequences present in the transcriptomes of *Pa* and *Paq*, respectively, only 2% (1297 transcripts) share a sequence identity higher than 95%. Modern ferns diversified into the Pteridoids (including *Paq*) and Eupolypoids (including *Pa*) 140 million years ago (Fig. 2b)^43^. A blast search of the *FOS1* gene sequence against the 68 fern transcriptomes available in the oneKP identified the presence of an identical transcript in *Phlebodium pseudoaureum*, a close relative to *Pa*. Deep mining of the transcriptomic resources documented the presence of homologous sequences across the evolutionary gap between these species (Supplementary Tables 2 and 3). If a common ancestral *FOS1* sequence was present in a progenitor to the derived ferns, the sequence has been under a remarkably high selection pressure. The four-electron oxidative decarboxylation reaction catalyzed by FOS1 is complex and may impose such a selection pressure. However, FMOs are “loaded guns” with the energy to drive an oxygenation reaction stored in the enzyme without precise docking of the substrate^33,39^. This can explain why *N*-hydroxyphenylalanine escapes the entire enzymatic reaction sequence and is present as an in vivo metabolite in *Pa*. *N*-hydroxyphenylalanine may have yet unrecognized functional roles in addition to being the initial intermediate in cyanogenic glycoside synthesis. The CYP79 family enzymes catalyzing the same set of reactions in higher plants show less sequence conservation. This may be because electron donation is provided to CYP enzymes by a separate NADPH-dependent cytochrome P450-oxidoreductase^3,44^. In CYP79-catalyzed reactions, all intermediates are bound within the active site as demonstrated by stable isotope experiments preventing release of the N-hydroxy amino acid intermediate^45^. Alternatively, the high sequence similarity between FOS1 from the two distantly related fern species may also reflect horizontal gene transfer. This phenomenon has been observed several times in fern species^46,47^. Ferns and other seed‐free plants may be more prone to horizontal gene transfer due to the weaker protection of the gametophytic eggs and sperm to the external environment, enabling transfer of genetic material^48^. Identification of the oxime-producing step in the cyanogenic fern species that phylogenetically lie within the 140 million years gap between *Pa* and *Paq* would establish the evolutionary relationships of these two ortholog genes.

**Fig. 7:**
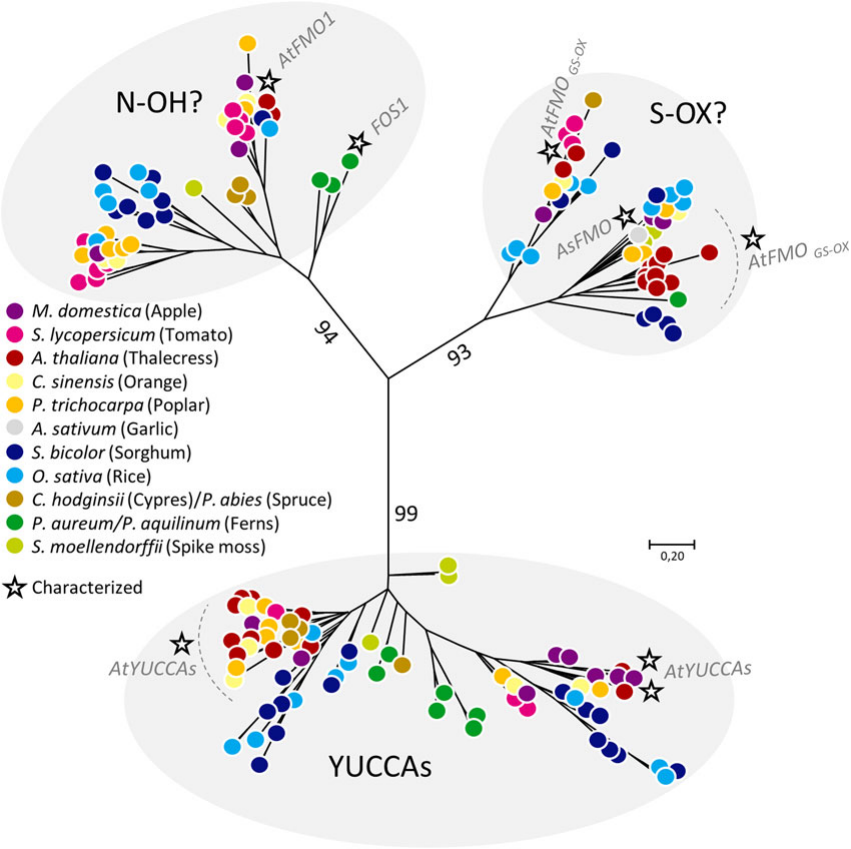
Phylogenetic tree of the flavin monooxygenase (FMO) superfamily containing all predicted full-length FMOs from ferns *(P. aureum and P. aquilinum)* together with FMOs from eight higher plant species. As all species are represented in each of the tree clades, the phylogenetic analysis suggests an early diversification of the groups prior to species differentiation. Employed sequence IDs are compiled in Supplementary Data 2. Characterized FMOs (or clusters of all characterized FMOs such as the Arabidopsis YUCCAs) are indicated by a star.

Class B flavin-dependent monooxygenases are found in all kingdoms of life^35^. All plant and animal flavin-dependent monooxygenases belong to this Class B FMO type, with a single exception of a Baeyer–Villiger monooxygense found in moss (*Physcomitrella patens*)^33,49^. The *Arabidopsis thaliana* genome contains 29 FMO genes^32,33^ and the human genome contains a gene cluster encoding five FMOs (FMO1-FMO5)^50^. The Pfam 31.0 database (accessed May 2020) lists a total of 1861 predicted Class B FMO sequences from 85 plant species (pfam.xfam.org^51^). To investigate the evolutionary diversity of plant FMOs, a representative set of FMO sequences from species representing land plant evolution, including the predicted full-length FMOs from fern transcriptomes (six from *Pa* and five from *Paq*, (Supplementary Fig. 6) and conifer (*Chamaecyparis hodginsii* and *Picea abies*) were used to build a phylogenetic tree (Fig. 7, Supplementary Table 4). The phylogenetic analysis includes representative functionally characterized FMOs: *As*FMO1 from garlic (*A. sativum*) catalyzing S-oxygenation of allyl-mercaptan^40^, the *A. thaliana* YUCCAs involved in the biosynthesis of the phytohormone auxin^52^, the *At*FMO_GS-OX1-5_ performing S-oxygenation of methylthioalkyl glucosinolates^53^, and *A. thaliana At*FMO1 that catalyzes N-hydroxylation of pipecolic acid to form *N*-hydroxypipecolic acid, the critical signaling molecule in systemic acquired resistance^54–56^. The analysis shows that plant FMOs cluster in three phylogenetically distinct groups (Fig. 7), with each harboring members from all evolutionary distinct species from *Selaginella* (moss) to *A. thaliana* (angiosperm). This suggests an evolutionary split of the FMOs prior to emergence of the early land plants.

By adding fern FMOs to a plant FMO-specific phylogenetic tree, the role of the other putative fern monooxygenases can be hypothesized (Fig. 2e, Supplementary Fig. 6). The analysis identified six transcripts, three orthologues from each fern species, to cluster in the YUCCA clade. To date all characterized YUCCAs are involved in auxin biosynthesis, as they are proposed to catalyze a decarboxylation of indole-3-pyruvate acid to form IAA. An additional role of thiol reductase activity has been linked to these enzymes^33,57^. The establishment of a YUCCA-like mediated auxin function for fern FMOs would contribute to the evolutionary perspectives of hormone biosynthesis and signaling. *Pa*FOS1 and *Paq*FOS1 group together with two additional FMO contigs (*Pa*22435 and *Paq*33416; Fig. 2e, Supplementary Fig. 6). This group of FMOs also encapsulates the *At*FMO1 catalyzing N-hydroxylation of pipecolic acid^54^ (Fig. 7). Based on the phylogeny and functional characterization, we suggest that the group encapsulating *Pa*FOS1, *Paq*FOS1, and *At*FMO1 catalyze N-hydroxylation reactions. The *Paq*FOS1 and the *At*FMO1 share 40% amino acid sequence identity (209/518) with a similarity score of 61% (318/518). As suggested by the SEC-elution profile, *Paq*FOS1 elutes solely as a homodimer. In parallel to *Paq*FOS1, we also expressed and isolated *At*FMO1 and demonstrated that it also elutes as a homodimer (Supplementary Fig. 7). A dimeric FMO protein has previously been isolated and crystallized from the methylotropic bacterium *Methylophaga*^58^. Most recently, the crystal structures and proposed dimeric arrangement of class B FMOs from multicellular organisms were reported for the pyrrolizidine alkaloid N-oxygenase (*Zv*PNO) from the Locust grasshopper (*Zonocerus variegatus*)^59^ and for ancestral reconstructed mammalian FMOs^60^. Based on the sequence similarities and crystal structures obtained, and in agreement with the isolation of *Paq*FOS1 and *At*FMO1 as stable homodimers, the tertiary structure of the class B FMOs are predicted to be conserved across the kingdoms of life.

Here, we show that an FMO catalyzing N-hydroxylation of an α-amino acid plays a key role in the convergent evolution of cyanogenic glycoside biosynthesis in ferns and higher plants. In all currently investigated gymnosperms and angiosperms, oxime formation from amino acids is catalyzed by a cytochrome P450 from the CYP79 family^18^. Our study demonstrates that the introduction of cyanogenic glycoside biosynthesis in ferns was based on independent recruitment of a unique class B FMO protein.

In addition to lacking a CYP79, ferns also lack the other cyanogenic glycoside-related CYP families: CYP71, CYP736, and CYP706 (Fig. 3). These families are all members of the large 71 clan, and selected members of these families catalyze conversion of oximes into the cyanohydrin intermediate in seed plants (Fig. 1). This opens speculation on the possible identity of the remaining biosynthetic pathway members in ferns. Based on less than 40% amino acid sequence identity criteria, many of the CYPs identified in the *Pa* and *Paq* transcriptomes did not correspond to any previously named and characterized P450 families. This study therefore unmasks a treasure trove of what is to our knowledge new CYP families and possible pathway candidates. Interestingly, other families of the 71 clan are also highly abundant in fern species^61^, and would be possible targets for gene discovery. The diversity of previously unnamed CYP families accentuates convergent evolution of cyanogenic glycoside biosynthesis as well as other metabolic pathways in ferns. Our comparative transcriptomic strategy to identify *FOS1* was highly successful, and would provide a robust approach to identify the remaining pathway members.

The evolution of the cyanogenic glycoside pathway is quite dynamic^12^. Recently, the classical three-step pathway was revised, with the discovery that sugar gum (*Eucalyptus cladocalyx*) harbors four genes that catalyze the conversion of amino acid to cyanogenic glycoside^10^. It is therefore also a possibility that more (or less) biosynthetic steps and intermediates might be present in ferns. Further, the cyanogenic glycoside biosynthetic pathway is shown to act as a dynamic metabolon, ensuring channeling of intermediates^62^. Here, *Sorghum bicolor* metabolons encounter the soluble UGT into tight organization. A likewise orchestration of FOS1 into a membrane-bound complex could be plausible, and indeed FMO from humans are associated with the membrane^50,60,63^. The identification of FOS1 in cyanogenic ferns alters traditional perceptions of the origin of cyanogenic glycoside biosynthesis, and highlights the importance of N-hydroxy amino acids, oximes, and cyanogenic glycosides throughout plant evolution.

At present, only a few plant FMOs have been functionally characterized. These FMOs have been shown to catalyze unique and crucial oxygenation reactions in plant hormone metabolism, pathogen resistance, signaling and chemical defense^32^. Our study demonstrates that the N-hydroxylating capacity of plant FMOs participate in the direct synthesis of a plant defense compound, and possible formation of undiscovered new metabolites via an N-hydroxy amino acid intermediate. Furthermore, this example of convergent evolution by the recruitment of different enzyme families—specifically showing that a soluble FMO can catalyze the same reaction as a membrane-bound cytochrome P450—opens opportunities for industrial applications in the future.

## Methods

### Plant material

Plant material for metabolite and RNA extraction was obtained from *Pa* (previously *Polypodium aureum*) grown in glass house at the Botanical Garden of Copenhagen (Plant ID E615) and from *Pteridium aquilinum* (*Paq*) collected at Dronningens Bøge, Esrum Lake, Nødebo, on June 17, 2015 (55°59′49.9″N, 12°21′27.6″E). Tissues were snap-frozen in liquid nitrogen and stored at −80 °C until further analyses.

### Metabolite profiling of fern tissue

Plant tissue (~30 mg) was weighed and boiled in 85% methanol (v/v, 300 μL) for 5 min. The vial was transferred to an ice bath and the material macerated with a small pestle. The supernatant obtained after centrifugation (13,000 × *g*, 1 min) was filtered (0.45 μm low-binding Durapore membrane) and diluted 1:5 in water prior to LC–MS analysis.

Analytical LC–MS was carried out using an Agilent 1100 Series LC (Agilent Technologies, Germany) coupled to an HCT Ultra ion trap mass spectrometer running in positive electrospray ionization (ESI) ultra-scan mode (Bruker, Bremen, Germany). The LC was fitted with a Zorbax SB-C18 column (2.1 × 50 mm, 1.8 μm; Agilent Technologies) and operated at 35 °C, with a flow rate of 0.2 mL min^−1^. The mobile phases were: (a) 0.1% HCOOH (v/v) and 50 μM NaCl; and (b) 0.1% HCOOH in MeCN (v/v). The gradient program was: 0–0.5 min, isocratic phase 2% B; 0.5–7.5 min, linear gradient 2–40% B; 7.5–8.5 min, linear gradient 40–90% B; 8.5–11.5 min, isocratic phase 90% B; 11.5–18 min, isocratic phase 2% B. The flow rate was raised to 0.3 mLmin ^−1^ in the interval 11.2–13.5 min. Traces of total ion current and of extracted ion currents for specific [M+Na]^+^ and [M+H]^+^ adduct ions were used to identify the eluted constituents using Compass DataAnalysis software (version 4.2, Bruker Daltonics). See below for targeted analytical LC–MS analysis of *N*-hydroxyphenylalanine from *Pa* tissue.

### RNA isolation and transcriptome mining

Total RNA was prepared from 30 to 50 mg of plant tissue using the Spectrum Plant Total RNA Kit (Sigma-Aldrich, US). Transcriptomes were prepared from mRNA isolated from *Pa* fiddlehead, *Pa* young frond, and from two *Paq* frond tips containing high and low cyanogenic glycoside levels. Transcriptome sequencing was carried out by Macrogen (Seoul, South Korea) using an Illumina HiSeq2000 sequencer (Illumina, San Diego, CA) to generate paired-end libraries. The reads were de novo assembled and relative transcript abundance estimated by Sequentia Biotech (www.sequentiabiotech.com) using the Trinity pipeline^64^.

The expression level of all transcripts was quantified. Identification of gene families was performed using the OrthoMCL pipeline (http://orthomcl.org/orthomcl/about.do). Differential gene expression analyses based on the *Pa* and *Paq* transcriptomes were carried out using eXpress^65^ and the data transferred into R and analyzed with the package NOISeq^66^.

### Isolation and transient expression of FMO candidate genes

The full-length sequence of the predicted ORF *Paq*18302 encoding gene (most upstream methionine) without codon optimization was fitted with attb1 and attb2 Gateway cloning sites: attB1: ggggacaagtttgtacaaaaaagcaggct, attB2: ggggaccactttgtacaagaaagctgggt and synthesized by GenScript. Guided by *Pa*22758, we isolated the full length of the FMO sequence from *Pa* cDNA using gene specific primers flanked by gateway sites (Forward: ggggaccactttgtacaagaaagctgggtctattcatctttgtagtccatgtta, Reverse: ggggaccactttgtacaagaaagctgggtctattcatctttgtagtccatgtta) and by PCR attached the gateway sites. The fragment was cloned into pUC57. Both constructs were subcloned by Gateway recombination from the pUC57 vector into the expression vector pEAQ3-HT-DEST^67^.

Cells from overnight cultures of *Agrobacterium tumefaciens* (AGL1) containing expression constructs with the target gene sequence under the control of CamV-35S promoter/terminator elements in pEAQ (*Paq*FOS1) or pJAM1502 *(CYP71AN24, UGT85A19*) or the gene sequence for the gene-silencing inhibitor protein p19^68,69^ were harvested by centrifugation (4000 × *g*, 10 min) and resuspended to OD_600_ 0.8 in water. After 1 h incubation at ambient temperature, the *A. tumefaciens* cultures were used to co-infiltrate leaves of 3–4 weeks old *Nicotiana benthamiana* plants. After 4–5 days, leaf discs (1 cm diameter) were excised from infiltrated leaves, frozen in liquid nitrogen, and subsequently ground and extracted in 200 μL 85% MeOH (v/v) for metabolite profiling as described above. Analytical LC–MS of extracts of infiltrated leaves was carried out either as described above using the ion trap instrument or using a Dionex Ultimate 3000 RS UHPLC (Thermo Fisher Scientific) system with DAD detector and fitted with a Phenomenex Kinetex^®^ XB-C18 column (1.7 μm, 100 × 21 mm; Phenomenex, US) operated at 40 °C and with a flow rate of 0.3 mL/min. The mobile phases were: (a) 0.1% HCOOH (v/v) and 50 mM NaCl; and (b) 0.1% HCOOH in MeCN (v/v). The gradient program: 0–1 min, isocratic gradient 5% B; 1–7 min, linear gradient 5%–70%; 7–8 min, linear gradient 70–100%; 8–10 min isocratic 100% B; 10–11 min, linear gradient 100–5% B; 11–16 min, isocratic 5% B. The UHPLC system was coupled to a compact™ qToF (Bruker Daltonics) mass spectrometer run in negative ESI mode from 50 to 1200 *m*/*z*. Raw data were processed using Compass DataAnalysis software (version 4.2, Bruker Daltonics).

### Generation of expression plasmids for *paq*FOS1 and *At*FMO1

The nucleotide sequence of contig *Paq18305* and of *AtFMO1* from *A. thaliana* (Q9LMA1) was codon optimized for expression in *E. coli* fitted with an upstream 6xHis encoding tag and inserted into the expression vector pET-30a(+). To express the recombinant proteins, both pET-30a-*Paq*FOS1 and pET-30a-*At*FMO1 were transformed into Phage-resistant BL21(DE3)-R3 strain (Structural Genomics Consortium Oxford).

### Expression and isolation of recombinant *Paq*FOS1 in *E. coli*

A single colony from a fresh plate (1.5% LB-agar containing 50 µg/mL kanamycin and 25 µg/mL chloramphenicol) was grown O/N at 37 °C with shaking (250 rpm) in 250 mL LB medium supplemented with kanamycin and chloramphenicol. A 4 L expression batch was set up by inoculation each liter of LB medium (supplemented with kanamycin and chloramphenicol) with 20 mL of bacterial culture into four 2.5 L Ultra Yield flasks fitted with AirOtop enhanced seals (Thomson Instrument, Germany). Preinduction cultures were incubated at 37 °C and 225 rpm to reach OD 1.2–1.5 before induction with IPTG (final concentration 0.5 mM). The expression culture was grown O/N at 18 °C and the *E. coli* cells sedimented (5000 × *g*, 10 min) and stored at −20 °C until used.

*E. coli* cells were thawed and resuspended in lysis buffer (1 g cells per 5 mL buffer; 100 mM HEPES pH 7.5, 500 mM NaCl, 1 mM MgSO_4_, 0.5 mM TCEP, Benzonase (25 U/mL; Sigma-Aldrich)) and lysed in a high-pressure homogenizer (Avestin EmulsiFlex D20, 40 psi). The lysate was clarified by centrifugation (11,000 rpm, 40 min), filtered (0.22 μM) and applied to two 5-mL His-Trap FF columns (GE Healthcare) connected in line and preequilibrated with Binding buffer (50 mM HEPES pH 7.5, 500 mM NaCl, 10 mM Imidazole, 0.5 mM TCEP). Columns were washed with ten column volumes of wash buffer (50 mM HEPES pH 7.5, 500 mM NaCl, 30 mM Imidazole, 0.5 mM TCEP) and protein eluted using a ten column volume 0–100% gradient of elution Buffer (50 mM HEPES pH 7.5, 500 mM NaCl, 500 mM Imidazole, 0.5 mM TCEP). The eluate was collected in 1.5 mL fractions in 96-wells plates. Fractions of interest were analyzed by SDS-PAGE using 4–12% NuPAGE gradient protein gels (Thermo Scientific). Fractions containing the target protein were concentrated to a final total volume of 5 mL by centrifugation using preequilibrated membrane filters (30 kDa cut off; Thermo scientific) and applied to a HiLoad 16/60 Superdex 200 column (120 mL, GE Healthcare). Before usage, columns were calibrated using the High molecular weight calibration kit (ranging from 43 to 669 kDa; GE Healthcare) and preequilibrated using gel filtration (GF) buffer (50 mM HEPES pH 7.5, 150 mM NaCl, 0.5 mM TCEP), which was also used for eluting the protein. All buffers, except the GF buffer, contained one tablet cOmplete protease inhibitor cocktail pr. Fifty milliliters of buffer (cOmplete Inhibitor, EDTA-free, Roche). The isolated protein was frozen in liquid nitrogen and stored at −80 °C.

### Recombinant enzyme assays

The activity of the isolated recombinant FOS1 protein was determined in vitro in assay mixtures (total volume: 50 µL) containing 30 µL of diluted FOS1 protein (0.345 mg mL^−1^) reconstituted with FAD (10 µM final concentration) and phenylalanine (50 µM final concentration). Enzyme reaction was initiated by addition of NADPH (final concentration 6 mM). Following incubation (1 h, 30 °C, 300 rpm), the reaction was stopped by addition of 100 µL MeOH. Assays without enzyme, substrate, or cofactor served as controls. All assays were carried out in triplicates.

### Analysis of recombinant *Paq*FOS1 and *Pa* tissue

Enzyme reaction mixtures (50 µL aliquots) were diluted with 50 µL Milli-Q grade water and filtered (Durapore^®^ 0.22 μm PVDF filter plates, Merck Millipore, Tullagreen, Ireland) together with filtered and diluted MeOH extracts of *Pa* fiddlehead and young pinna (see above). Samples were chromatographically separated using an Advance UHPLC system (Bruker, Bremen, Germany) fitted with a Zorbax Eclipse XDB-C18 column (100 × 3.0 mm, 1.8 µm, Agilent Technologies, Germany) with a column temperature maintained at 40 °C. The mobile phases were: (a) HCOOH (0.05%, v/v); and (b) MeCN in 0.05% (v/v) HCOOH. The gradient elution profile was: 0–0.5 min, isocratic phase 3% B; 0.5–3.8 min, linear gradient 3–70% B; 3.8–4.4 min. linear gradient 70–100% B; 4.4–4.9 min, isocratic phase 100% B, 4.9–5.0 min, linear gradient 100–3% B; 5.0–6.0 min, isocratic phase 3% B using a flow rate of 0.5 mL min^−1^. The EVOQ Elite Triplequadrupole mass spectrometer (Bruker, Bremen, Germany) was equipped with an ESI operated in positive mode. The instrument parameters were optimized by infusion experiments with pure standards. The ion spray voltage was maintained at +4000 V, cone temperature was set to 350 °C and cone gas to 20 psi. Heated probe temperature was set to 400 °C and probe gas flow to 50 psi. Nebulizing gas was set to 60 psi and collision gas to 1.6 mTorr. Nitrogen was used as probe and nebulizing gas, and argon as collision gas. Active exhaust was constantly on. Multiple reaction monitoring (MRM) was used to monitor analyte parent ion → product ion transitions. MRM transitions and collision energies were optimized by direct infusion experiments into the MS (Supplementary Table 4). Both Q1 and Q3 quadrupoles were maintained at unit resolution. Bruker MS Workstation software (Version 8.2.1, Bruker, Bremen, Germany) was used for data acquisition and processing.

### Phylogenetic analysis

FMO sequences from angiosperms and mosses were accessed from Phytozome (phytozome.jgi.doe.gov, version 12.1.6). The following eight species were selected to span the evolution of higher plants: *Malus domestica*, *Solanum lycopersicum*, *Arabidopsis thaliana*, *Citrus sinensis*, *Populus trichocarpa*, *Sorghum bicolor*, *Oryza sativa*, and *Selaginella moellendorffii*. Their FMO sequences were obtained using BLASTp 2.2.26+ with the *A. thaliana* FMOs as query sequences resulting in a total of 169 hits. Fern sequences were accessed from the transcriptomes of *Pa* and *Paq* reported in the present study (*Paq*- or *Pa*TRINITY). To obtain robust phylogenetics, only full-length sequences were included. In all cases the ORF and initial methionine were chosen. FMO sequences from conifers were obtained from the databases OneKP (ualberta.ca/onekp) and Congenie (congenie.org) by blast-searching *Chamaecyparis hodginsii* (four sequences) and *Picea abies* (four sequences) with Arabidopsis FMOs as query sequences. The functionally characterized S-oxygenating FMO (*As*FMO1) from garlic (*Allium sativum*) was also included^40,70^. Sequence analyses were conducted using MEGA 7.0. All amino acid sequences were manually inspected before being aligned using ClustalW. The phylogenetic relationship was inferred using the maximum likelihood method based on the JTT matrix-based model and *n* = 100 replicates for bootstrapping. The phylogenetic tree is drawn to scale, not rooted, and with branch lengths measured in the number of substitutions per site. The analysis involved 189 amino acid sequences. The sequence IDs employed in the phylogenetic analysis can be found in Supplementary Data 2.

### Small read archive (SRA) searches for *FOS1* orthologues

Data from the fern whole genome duplication study at Fudan University were retrieved from NCBI (accession PRJNA422112, date 12 December 2017). The SRA searches were carried out using *PaqFOS1* as query. An FMO hit was obtained from all but four of the 119 fern transcriptomes, using a conserved N-terminal fragment as query.

### Chemicals

[UL-^14^C]-l-phenylalanine (0.25 μCi, specific activity 487 mCi mmol^−1^) was purchased from Perkin-Elmer. *N*-hydroxyphenylalanine, (*E*)- and (*Z*)-phenylacetaldoxime and prunasin were chemically synthesized as previously reported^71–73^. Specifically, chemical synthesis of L-(*N*-hydroxy)phenylalanine is outlined in Supplementary Fig. 8. Vicianin was obtained from a methanol extract of *Vicia sativa* seeds. Compound validation was based on UV absorption and accurate mass upon LC–MS analysis. Quantification of vicianin was carried out using a dilution series of amygdalin as reference compound, as the diglycosides are structurally comparable and expected to behave similarly with respect to their degree of ionization and UV absorption.

### Statistics and reproducibility

The fern species were chosen based on published literature reports of cyanogenic glycoside content, and confirmed by LC–MS analysis. RNA was extracted from identified tissue at least twice, with the highest quality RNA used for downstream transcriptomic analysis. Functional characterization by agroinfiltration in *Nicotiana benthamiana* plants was repeated in three independent experiments, using two biological *N. benthamiana* replicates, and three technical replicates each time. Similarly, in vitro enzyme assays in *E. coli* were repeated in three independent experiments with three technical replicates each time. *FOS1* constructs expressed in *A. tumefaciens* and *E. coli* were confirmed by sequencing.

### Reporting summary

Further information on research design is available in the Nature Research Reporting Summary linked to this article.

## Supplementary information

Supplementary Information

Description of Additional Supplementary Items

Supplementary Data 1

Supplementary Data 2

Supplementary Data 3

Reporting Summary

## Data Availability

The OneKP database is accessible at (ualberta.ca/onekp). The data from 120 SRA experiments are residing at NCBI as the fern whole genome duplication study with accession PRJNA422112. All other data are available in the main text or in the supplementary materials (Supplementary Data 1–3). Sequence data from this article can be found in the EMBL/GenBank data libraries under accession numbers MT856954 and MT856955 for the fern oxime synthase *Paq*18302 and *Pa*22578.

## Code availability

Details about software and algorithms used in this study are given in the “Methods” section. No customized code or algorithm deemed central to the conclusion was used.
