# Supplementary material for: A flavin-dependent monooxygenase catalyzes the initial step in cyanogenic glycoside synthesis in ferns
Source: Commun Biol. 2020 Sep 11;3:507. doi: 10.1038/s42003-020-01224-5 (PMC7486406; doi:10.1038/s42003-020-01224-5)
Supplement: Supplementary file 1 — Supplementary Information [file 42003_2020_1224_MOESM1_ESM.docx]

**Supplementary information for the manuscript “A Flavin-dependent Monooxygenase Catalyzes the Initial Step in Cyanogenic Glucoside Synthesis in Ferns”**

**
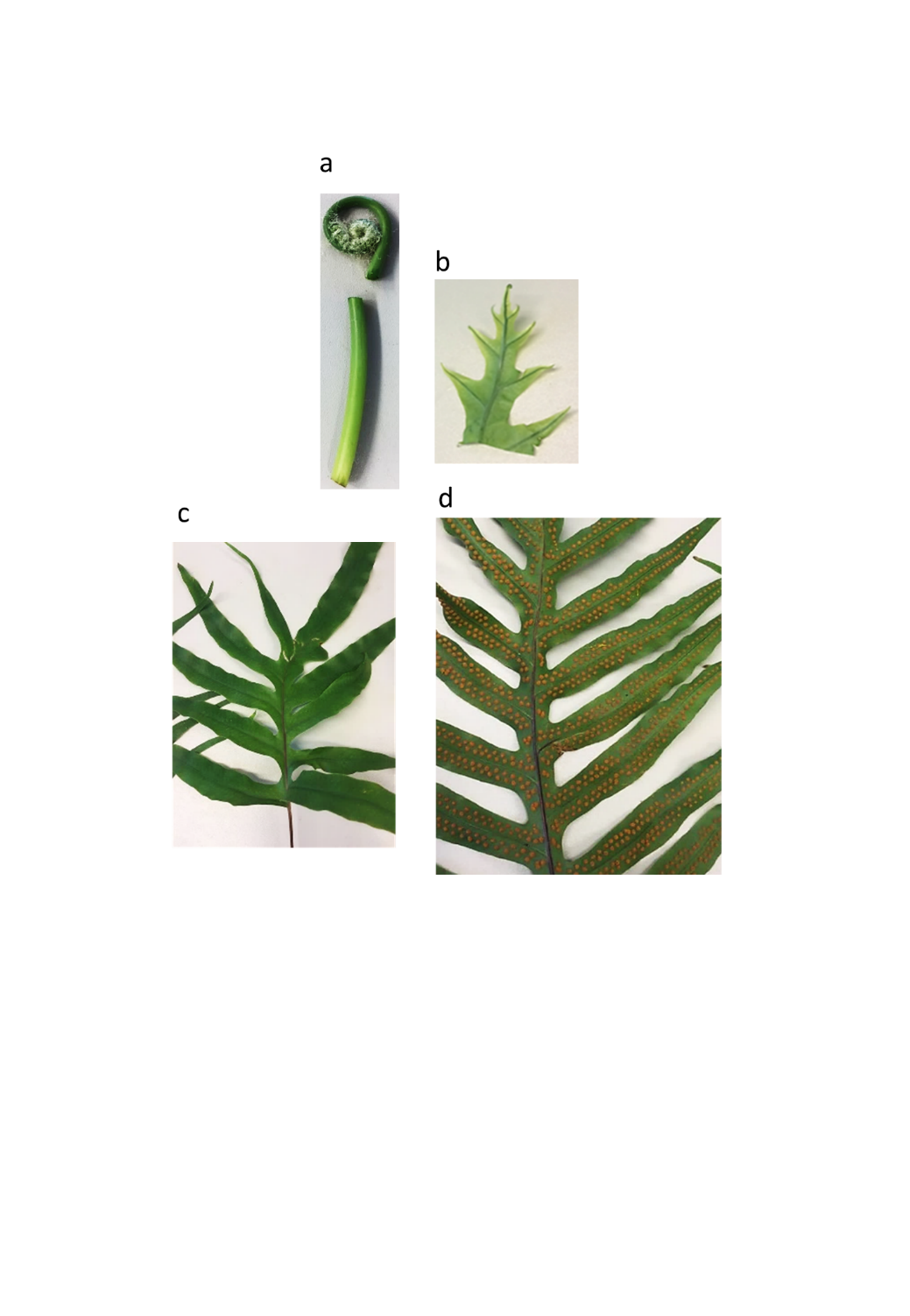
**

**Supplementary** **Fig. 1:** Tissue types of *Phlebodium aureum*. (a) Fiddlehead and stem (b) Young pinnae (c) Developing pinnae (d) Mature pinnae with spores. LC-MS analysis revealed that vicianin was the dominant cyanogenic glycoside in all tissues, although the mono-glucoside prunasin was also detected in much lower amounts (data not shown). Fiddlehead and young pinnae were selected for transcriptomic analysis.


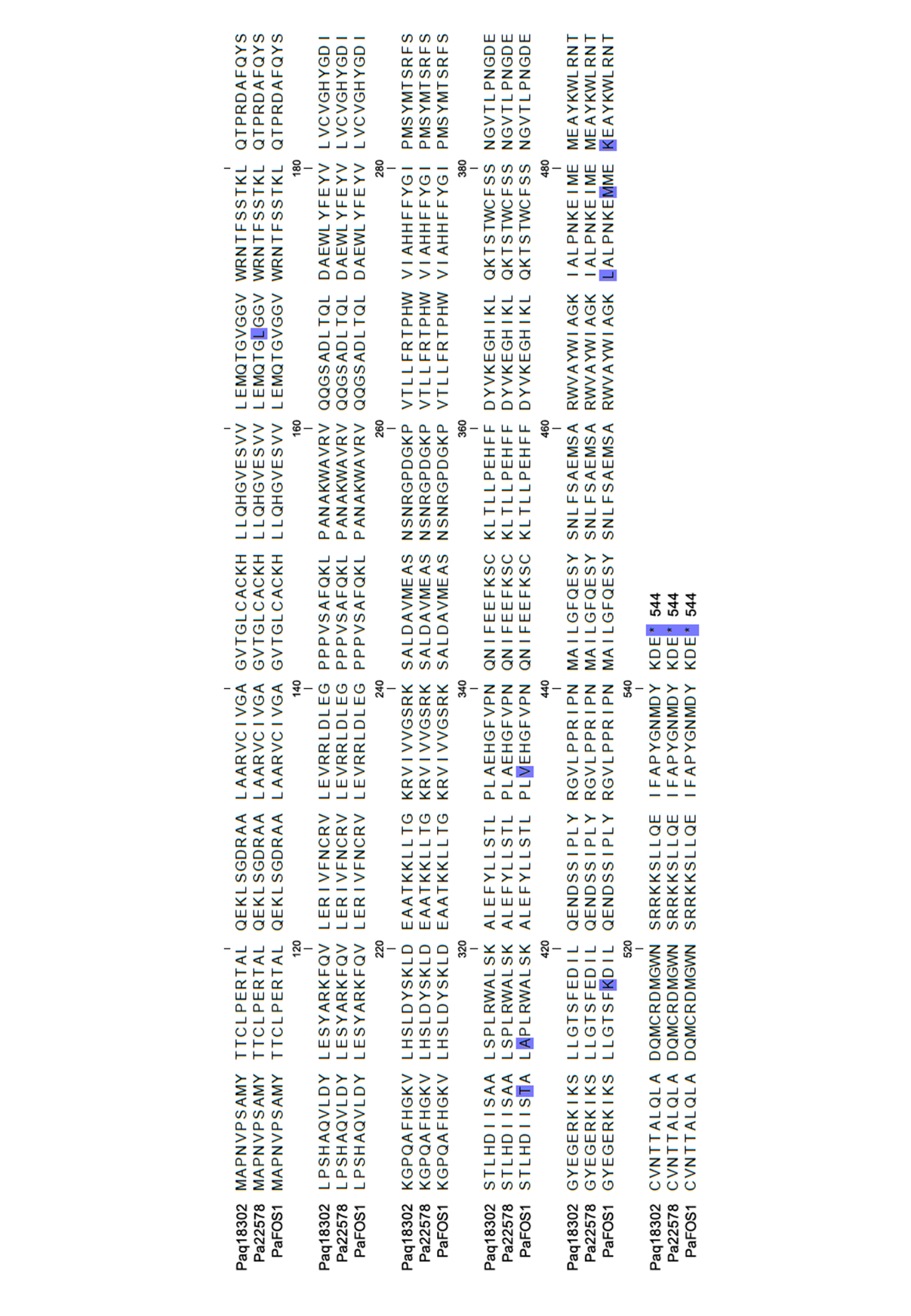


**Supplementary Fig. 2:** Sequence alignment of three FOS1 sequences: Contig *Paq*18302 from the transcriptome of *P. aquilinum* (referred in this work to as *Paq*FOS1), Contig *Pa*22758 from the transcriptome of *P. aureum. Pa*FOS1 is the isolated sequence from cDNA of *P. aureum* fiddlehead. Differences in the amino acid sequences are shown.


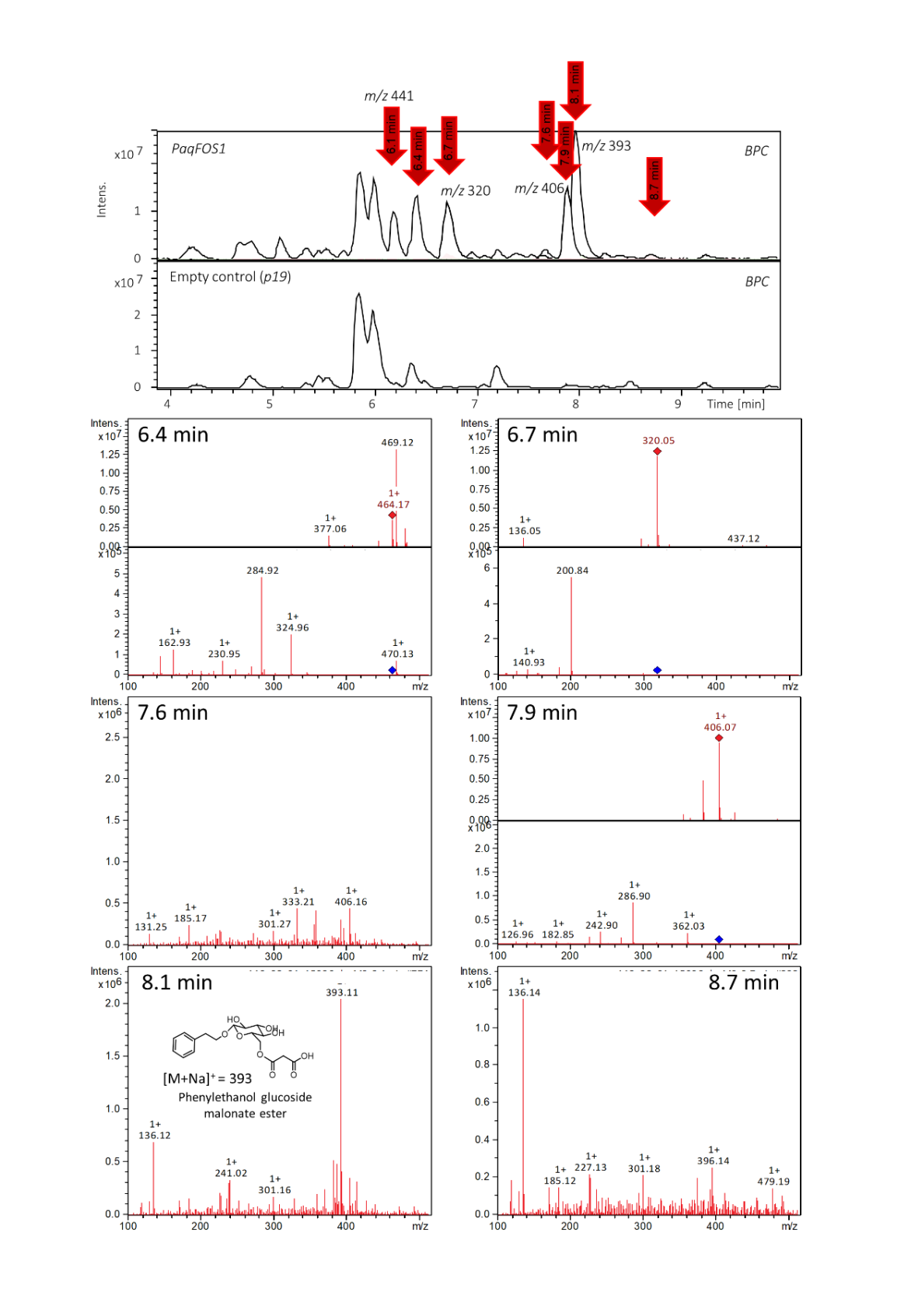


**Supplementary Fig. 3:** Metabolite analyses of *N. benthamiana* leaves transiently expressing *PaqFOS1*. Upper panel: Base Peak Chromatograms (BPCs) of the metabolite extracts from *N. benthamiana* leaves expressing *PaqFOS1* using expression of *p19* as an empty vector control.

Lower panels: The MS/MS profiles of six novel constituents (marked with red arrows in upper panel) formed upon expression of *PaqFOS1* in *N. benthamiana*:

*m/z* 320 at 6.7 min corresponding to the [M+Na]+ adduct of glucosylated phenylacetaldoxime;

*m/z* 406 at 7.6 and 7.9 min corresponding to the [M+Na]+ adduct of a glycosylated phenylacetaldoxime-malonic acid conjugate;

*m/z* 136 at 8.7 min corresponding to [M+Na]+ adduct of the phenylacetaldoxime;

*m/z* 393 at 8.1 min corresponding to [M+Na]+ adduct of phenylethanol glucoside malonate ester

*m/z* 441.08 and *m/z* 464.17 at 6.1 min and 8.1, respectively, are additional pathway-unrelated constituents formed upon transient expression in *N. benthamiana* leaves.


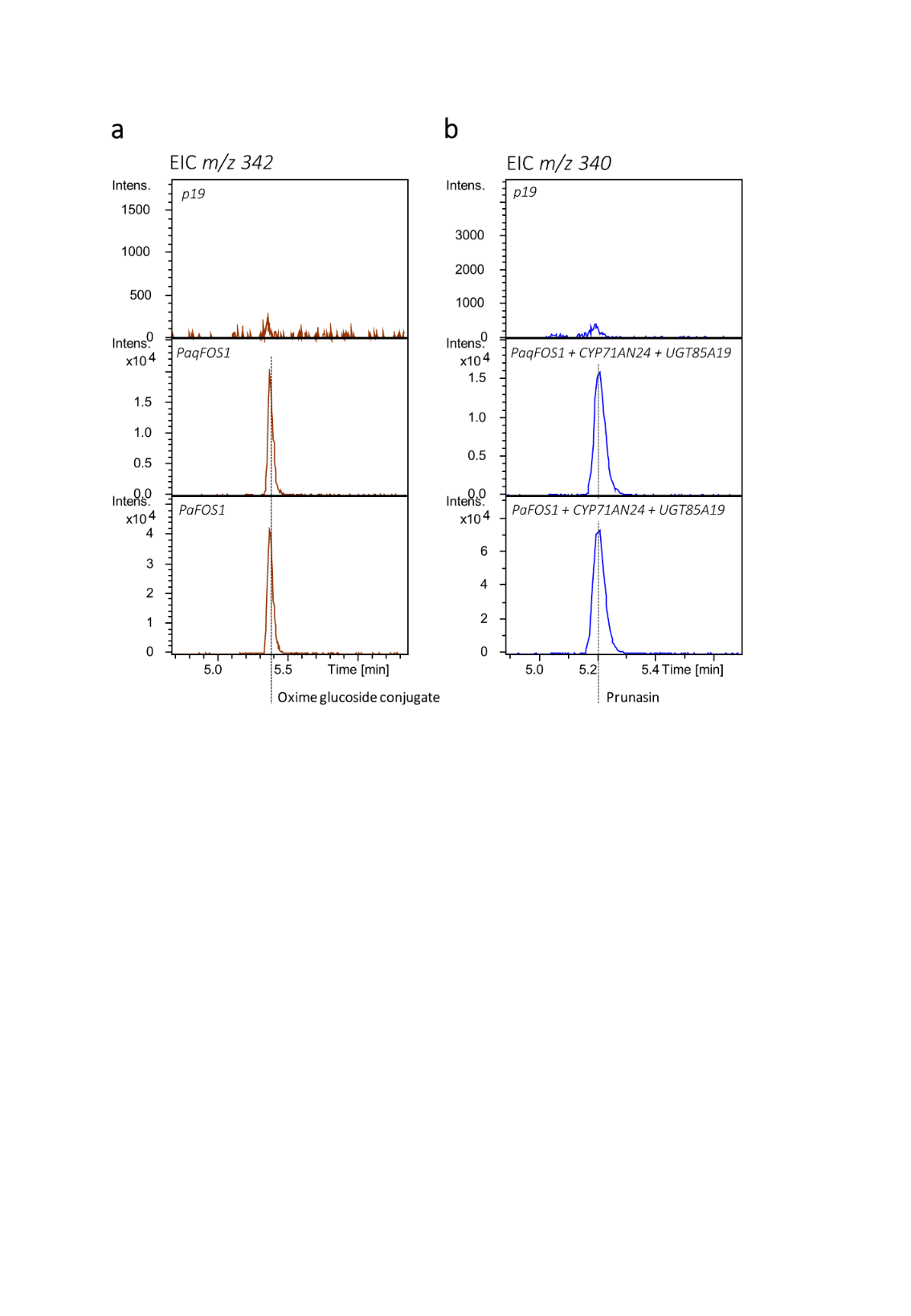


**Supplementary Fig. 4:** Extracted ion chromatograms (EICs) from LC-MS analysis of *N. benthamiana* metabolite extracts expressing *PaqFOS1* (*Pa22758*) or *PaFOS1* and following co-expression with *CYP71AN24* and *UGT85A19* and using expression of *p19* as a negative control. (a) EIC of *m/z* 340 [M-H2O+HCOOH]+ corresponding to phenylacetaldoxime-glucoside. (b) shows EIC of *m/z* 342 [M-H2O+HCOOH]+ corresponding to prunasin. Expression of FOS1s in combination with the two almond prunasin pathway genes *CYP71AN24* and *UGT85A19* produces prunasin eluting with a retention time of 5.25 min.


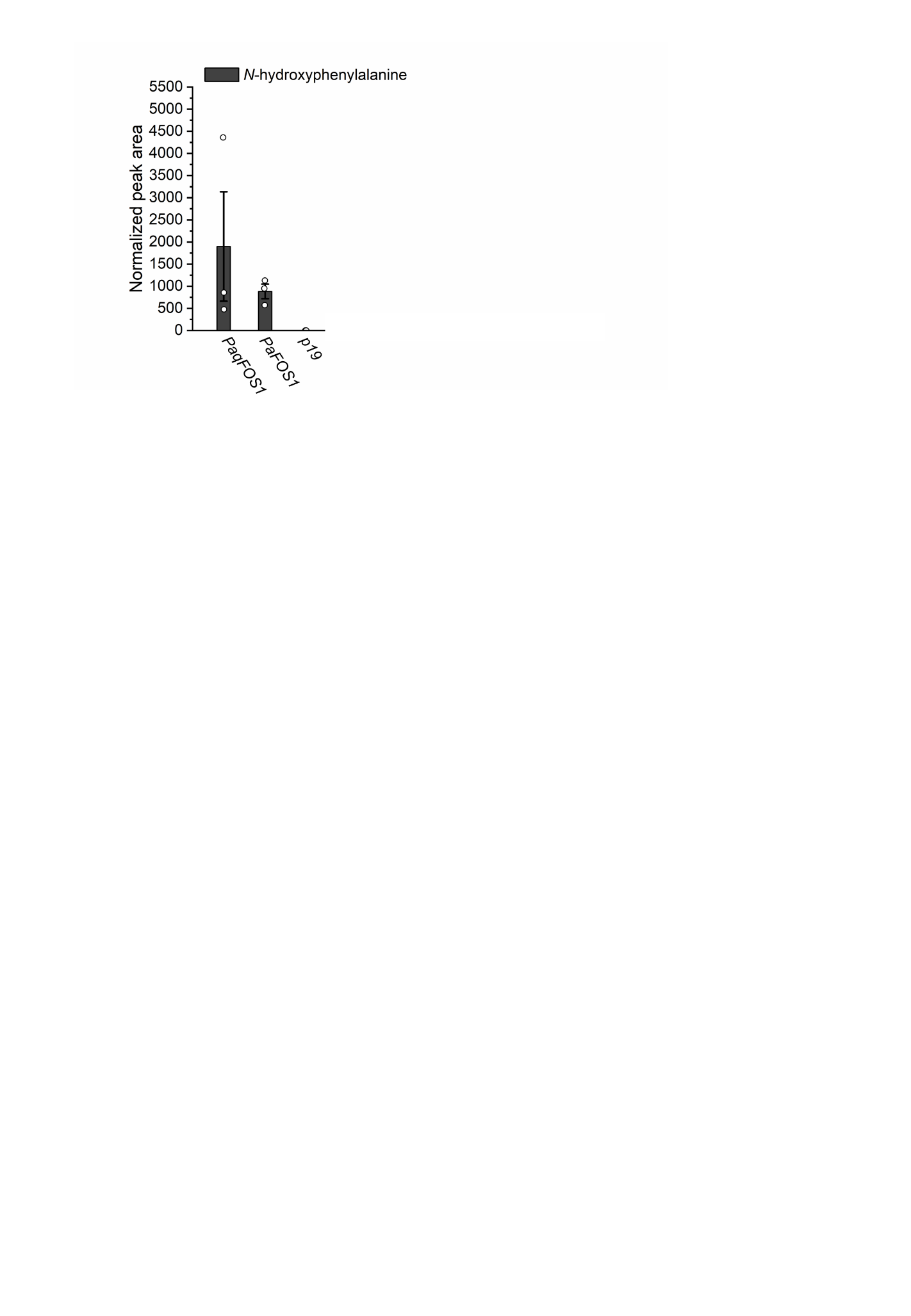


**Supplementary Fig. 5:** The presence of *N-*hydroxyphenylalanine in *N. benthamiana* leaves expressing *PaqFOS1* or *PaFOS1* detected by triplequadrupole LC-MS analysis of metabolite extracts*.* Expression of *p19* alone was used as negative control.


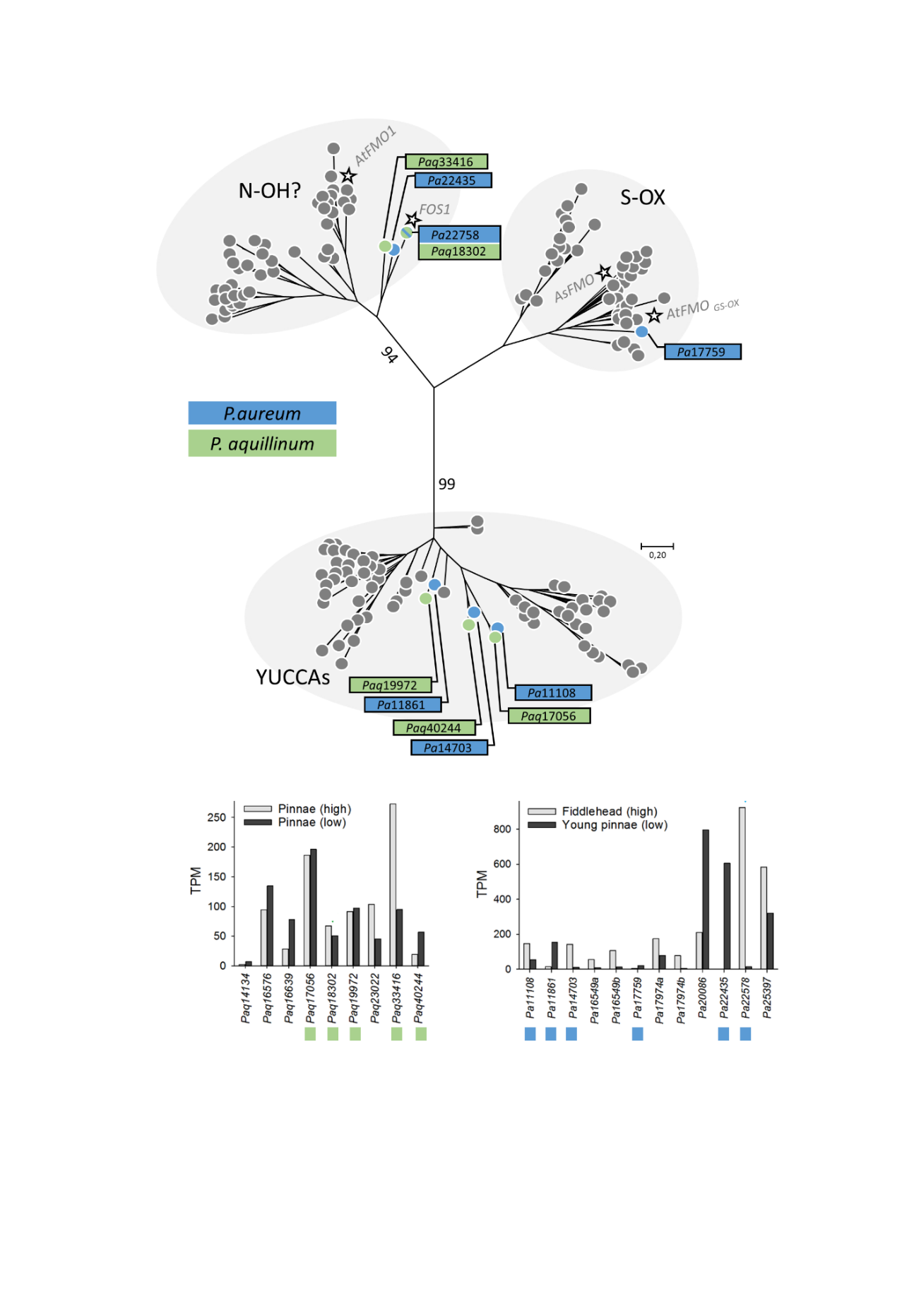


**Supplementary Fig. 6:** Phylogenetic tree of the Flavin-dependent monooxygenase (FMO) superfamily containing all predicted full-length FMOs from ferns (*P. aureum and P. aquilinum*) together with FMOs from eight higher plant species. The tree is identical to Fig 7, but encompasses full-length fern contigs identified in this study (Fig. 2e). Employed sequence IDs are compiled in Data S2.


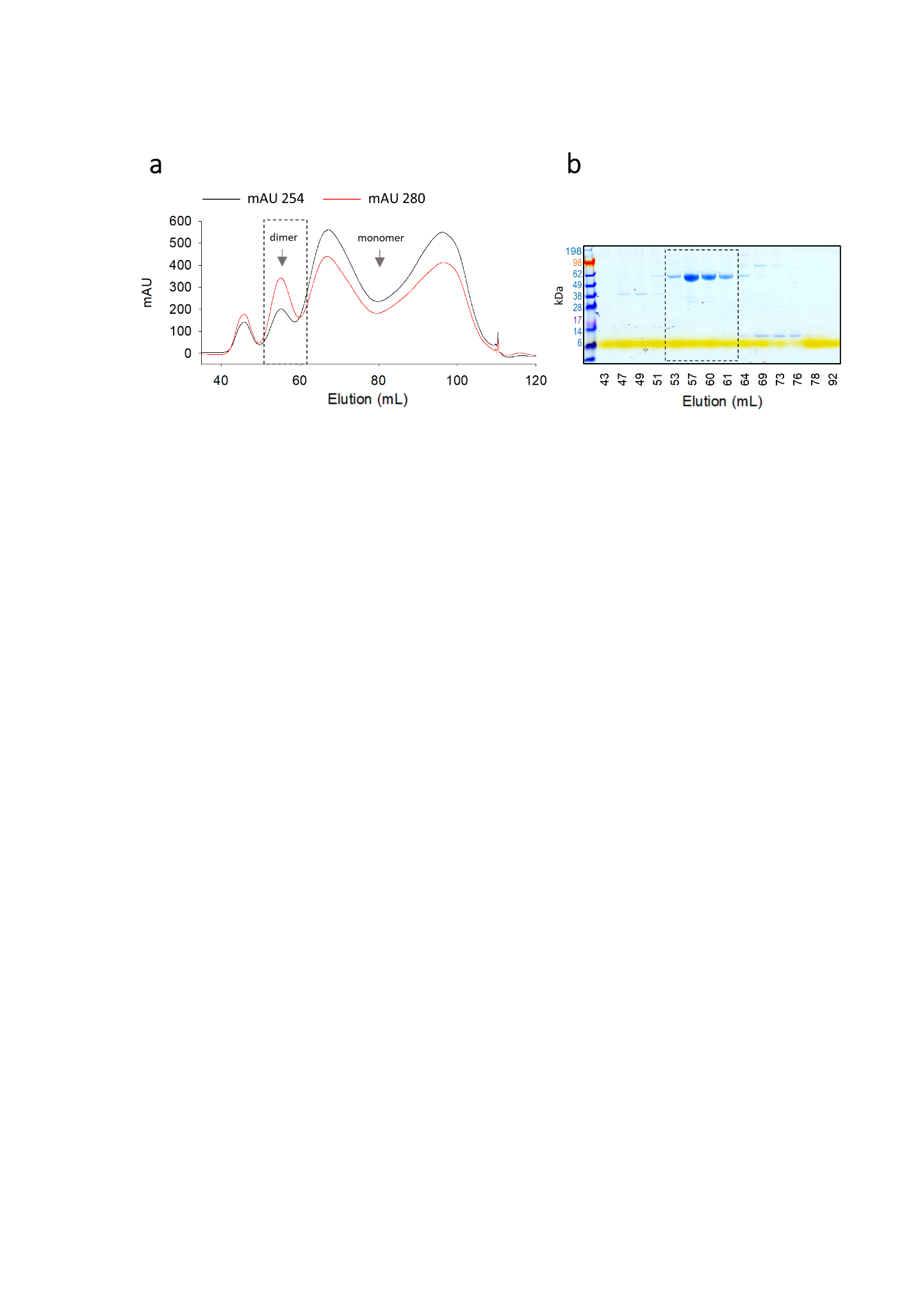


**Supplementary Fig. 7:** Size exclusion chromatography of the affinity purified AtFMO1 demonstrates that the protein is a homodimer. (a) The elution profile of the AtFMO1 upon gel filtration as monitored by the absorption at 280 nm (red) and 254 nm (black). (b) protein composition of the collected fractions following SDS-PAGE and Coomassie Brilliant Blue staining. The calculated mass of AtFMO1 is 61.2 kDa. AtFMO1 elutes as a single peak at a position calculated to correspond to a molecular mass of 120 kD corresponding to a homodimer.


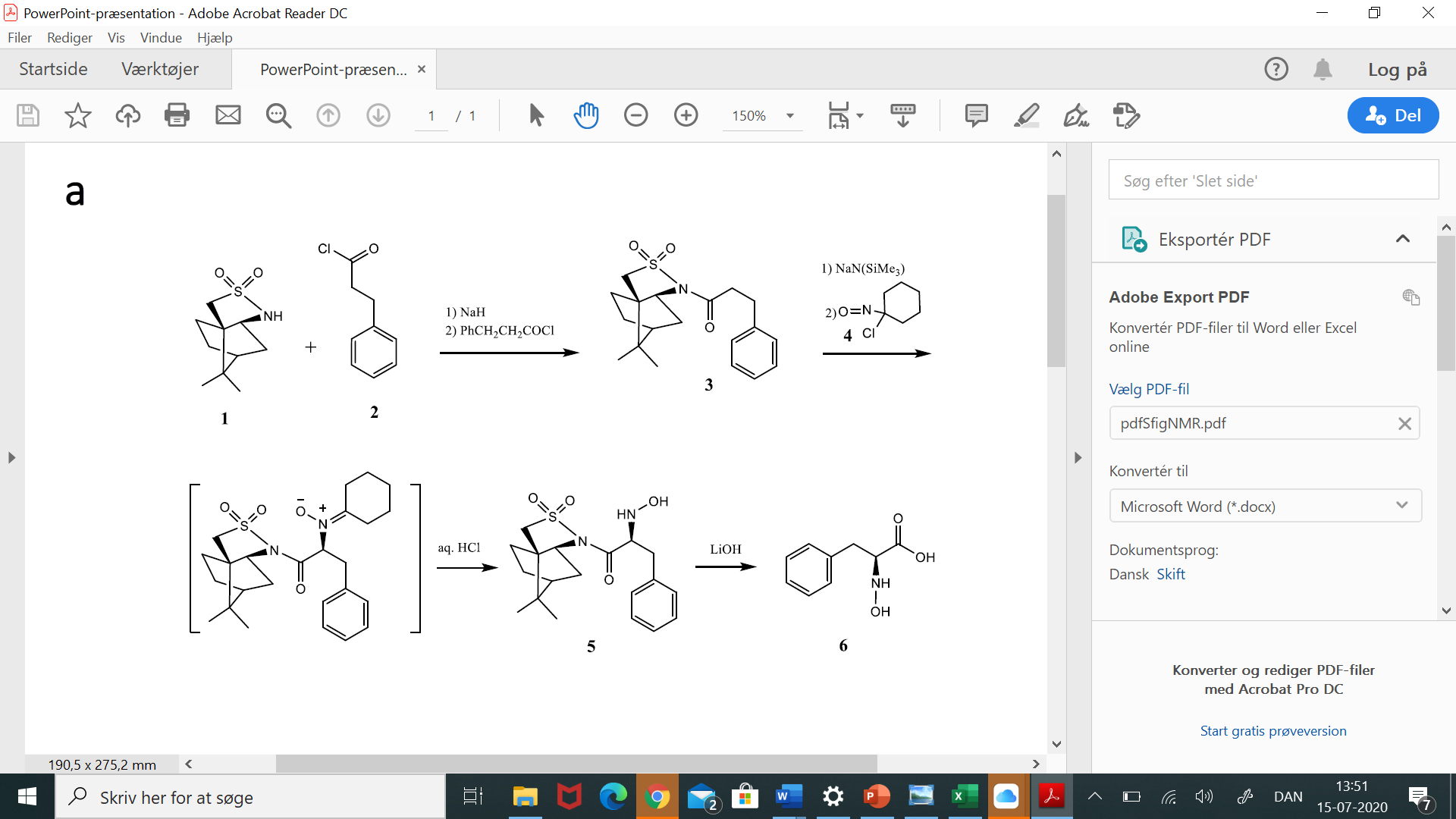


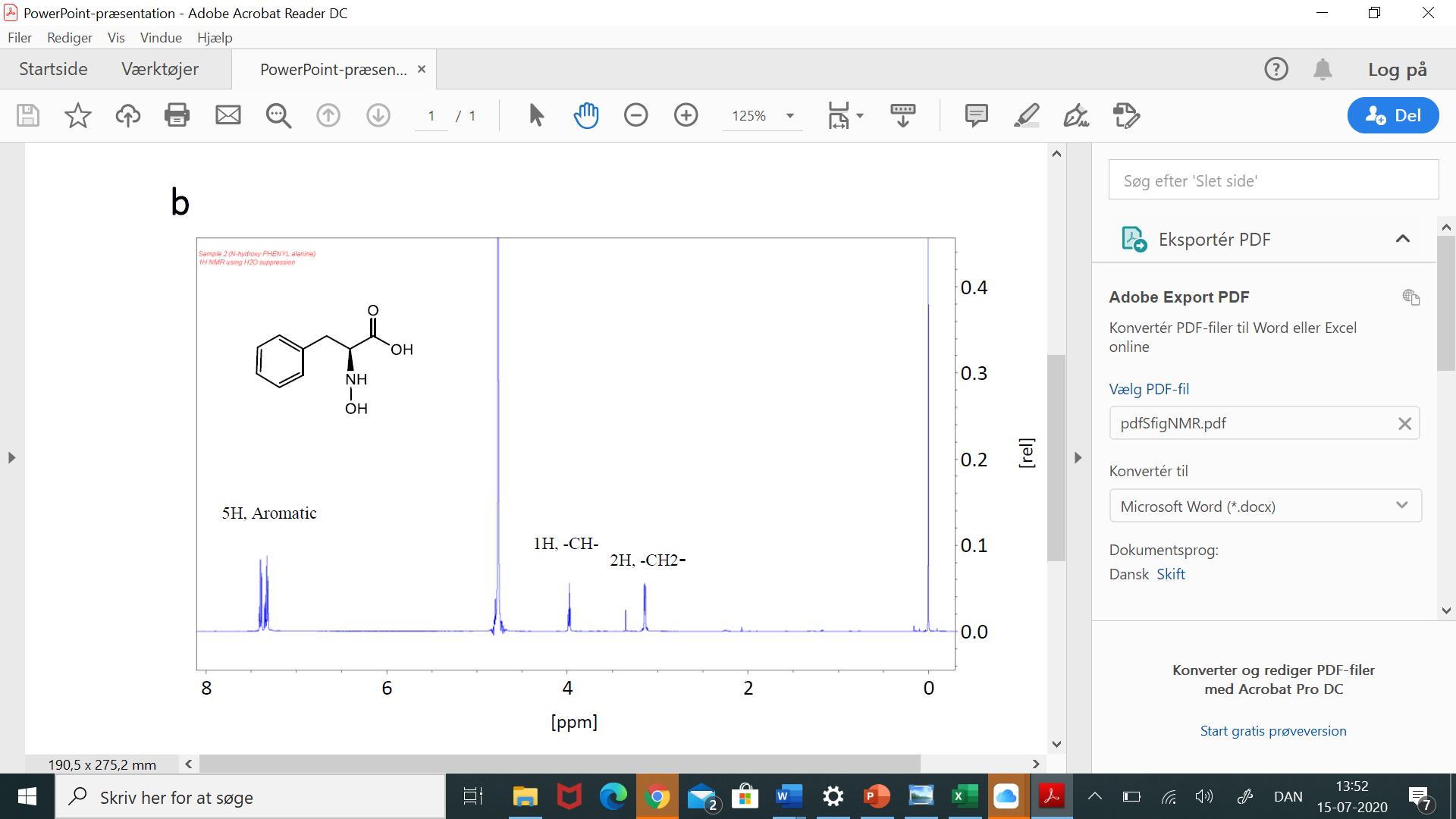


**Supplementary Fig. 8:** Chemical synthesis of *N*-hydroxyphenylalanine. (a) The commercially available (1*S*)-(-)-2,10-camphorsultam 1 was acylated with 3-phenylpropanoyl chloride to afford (2*S*)-*N*-(3-Phenylpropanoyl)bornane-10,2-sultam (**3**). Successive treatment of **3** with sodium hexamethyldisilazide-1-chloro-1-nitrosocyclohexane (**4**), and aq. HC1 gave pure, crystalline *N*-hydroxy-α-amino acid derivative (**5**). Mild saponification of **5** with LiOH afforded pure L-(*N*-hydroxy)phenylalanine (**6**) as shown from its 1H-NMR spectrum. (b): 1H-NMR (600 MHz, D2O) of the chemically synthesized L-(*N*-hydroxy)phenylalanine.

Supplementary Table 1.

| Family | *P. aureum* | *P. aquilinum* |
| --- | --- | --- |
| Cytochromes P450 (CYP) | 120 | 139 |
| Heme-binding protein | 5 | 5 |
| Berberine bridge enzyme (BBE) | 6 | 4 |
| Flavin monooxygenase (FMO) | 9(12) | 6(9) |

Number of potential monooxygenase encoding gene candidates present in the fern species *P. aureum* and *P. aquilinum*. The numbers in parenthesis include partial contigs that were not considered for further analysis.

Supplementary Table 2.

| **Species** | **SRX ID** | **Read Id(s)** | **% similar to FOS1** |
| --- | --- | --- | --- |
| [*Lindsaea heterophylla*](https://www.ncbi.nlm.nih.gov/Taxonomy/Browser/wwwtax.cgi?mode=Info&id=866197) | [SRX3868001](https://www.ncbi.nlm.nih.gov/sra/SRX3868001) | gnl\|SRA\|SRR6920724.7601586.2  gnl\|SRA\|SRR6920724.7601586.1 | 94 % |
| [*Osmolindsaea odorata*](https://www.ncbi.nlm.nih.gov/Taxonomy/Browser/wwwtax.cgi?mode=Info&id=32131) | SRX3868000 | gnl\|SRA\|SRR6920725.6188565.1 | 93 % |
| [*Rhachidosorus sp. XQ-2018*](https://www.ncbi.nlm.nih.gov/Taxonomy/Browser/wwwtax.cgi?mode=Info&id=2137828) | [SRX3868091](https://www.ncbi.nlm.nih.gov/sra/SRX3868091) | gnl\|SRA\|SRR6920634.4372284.2  gnl\|SRA\|SRR6920634.4372284.1 | 92 % |
| [*Lomariopsis boninensis*](https://www.ncbi.nlm.nih.gov/Taxonomy/Browser/wwwtax.cgi?mode=Info&id=2137822) | [SRX3868078](https://www.ncbi.nlm.nih.gov/sra/SRX3868078) | gnl\|SRA\|SRR6920647.11502369.2  gnl\|SRA\|SRR6920647.11502369.1 | 89 % |
| [*Hypodematium crenatum*](https://www.ncbi.nlm.nih.gov/Taxonomy/Browser/wwwtax.cgi?mode=Info&id=65731) | [SRX3868070](https://www.ncbi.nlm.nih.gov/sra/SRX3868070) | gnl\|SRA\|SRR6920655.1571079.2  gnl\|SRA\|SRR6920655.1571079.1 | 89 % |
| [*Rhachidosorus pulcher*](https://www.ncbi.nlm.nih.gov/Taxonomy/Browser/wwwtax.cgi?mode=Info&id=529618) | [SRX3868094](https://www.ncbi.nlm.nih.gov/sra/SRX3868094) | gnl\|SRA\|SRR6920631.16489087.2  gnl\|SRA\|SRR6920631.16489087.1 | 88 % |
| *[Stenochlaena](https://www.ncbi.nlm.nih.gov/Taxonomy/Browser/wwwtax.cgi?mode=Info&id=32079)* [*palustris*](https://www.ncbi.nlm.nih.gov/Taxonomy/Browser/wwwtax.cgi?mode=Info&id=32079) | [SRX3868112](https://www.ncbi.nlm.nih.gov/sra/SRX3868112) | gnl\|SRA\|SRR6920613.15041.1  gnl\|SRA\|SRR6920613.15041.2 | 88 % |
| [*Deparia petersenii*](https://www.ncbi.nlm.nih.gov/Taxonomy/Browser/wwwtax.cgi?mode=Info&id=65727) | [SRX3868114](https://www.ncbi.nlm.nih.gov/sra/SRX3868114) | gnl\|SRA\|SRR6920611.5125256.1  gnl\|SRA\|SRR6920611.5125256.2 | 88 % |
| [*Diplazium esculentum*](https://www.ncbi.nlm.nih.gov/Taxonomy/Browser/wwwtax.cgi?mode=Info&id=29615) | [SRX3868067](https://www.ncbi.nlm.nih.gov/sra/SRX3868067) | gnl\|SRA\|SRR6920658.3625264.1  gnl\|SRA\|SRR6920658.3625264.2 | 86 % |
| [*Prosaptia obliquata*](https://www.ncbi.nlm.nih.gov/Taxonomy/Browser/wwwtax.cgi?mode=Info&id=263117) | [SRX3868038](https://www.ncbi.nlm.nih.gov/sra/SRX3868038) | gnl\|SRA\|SRR6920687.1797433.2  gnl\|SRA\|SRR6920687.1797433.1 | 86 % |
| [*Loxogramme biformis*](https://www.ncbi.nlm.nih.gov/Taxonomy/Browser/wwwtax.cgi?mode=Info&id=2137823) | [SRX3868039](https://www.ncbi.nlm.nih.gov/sra/SRX3868039) | gnl\|SRA\|SRR6920686.10120512.1  gnl\|SRA\|SRR6920686.10120512.2 | 86 % |
| [*Pleocnemia winitii*](https://www.ncbi.nlm.nih.gov/Taxonomy/Browser/wwwtax.cgi?mode=Info&id=450142) | [SRX3868079](https://www.ncbi.nlm.nih.gov/sra/SRX3868079) | gnl\|SRA\|SRR6920646.1474362.2  gnl\|SRA\|SRR6920646.1474362.1 | 86 % |
| [*Angiopteris fokiensis*](https://www.ncbi.nlm.nih.gov/Taxonomy/Browser/wwwtax.cgi?mode=Info&id=397666) | [SRX3868110](https://www.ncbi.nlm.nih.gov/sra/SRX3868110) | gnl\|SRA\|SRR6920615.4826294.1  gnl\|SRA\|SRR6920615.4826294.2 | 86 % |
| [*Didymochlaena truncatula*](https://www.ncbi.nlm.nih.gov/Taxonomy/Browser/wwwtax.cgi?mode=Info&id=173906) | [SRX3868063](https://www.ncbi.nlm.nih.gov/sra/SRX3868063) | gnl\|SRA\|SRR6920662.11371198.1 | 86 % |
| [*Onoclea sensibilis*](https://www.ncbi.nlm.nih.gov/Taxonomy/Browser/wwwtax.cgi?mode=Info&id=3281) | [SRX3868115](https://www.ncbi.nlm.nih.gov/sra/SRX3868115) | gnl\|SRA\|SRR6920610.20440123.1  gnl\|SRA\|SRR6920610.20440123.2 | 86 % |
| [*Odontosoria chinensis*](https://www.ncbi.nlm.nih.gov/Taxonomy/Browser/wwwtax.cgi?mode=Info&id=32133) | [SRX3868010](https://www.ncbi.nlm.nih.gov/sra/SRX3868010) | gnl\|SRA\|SRR6920715.17710010.1  gnl\|SRA\|SRR6920715.17710010.2 | 85 % |

Scan of transcriptomes from 111 different fern species for the presence of FMOs with high sequence identity to FOS1 using the N-terminal region of *Paq*FOS1 (RVCIVGAGVTGLCACKHLLQH) as the bait. Fern species with a sequence identity above 85 % are listed.

Supplementary Table 3. *
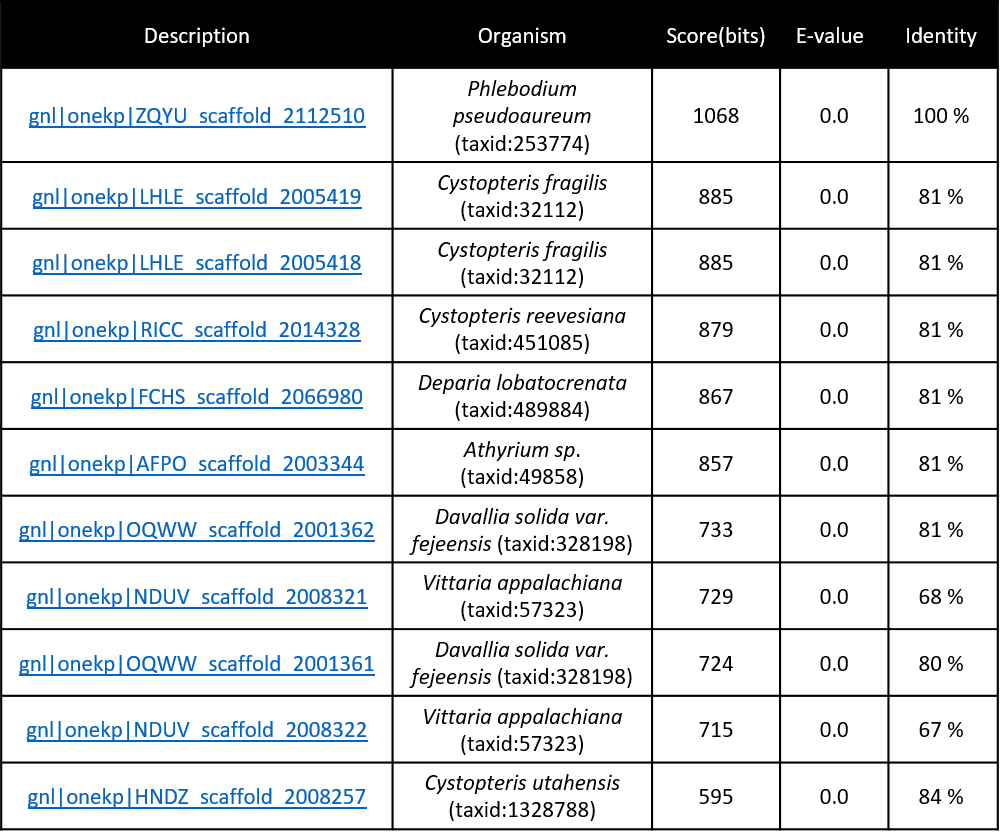
*

*
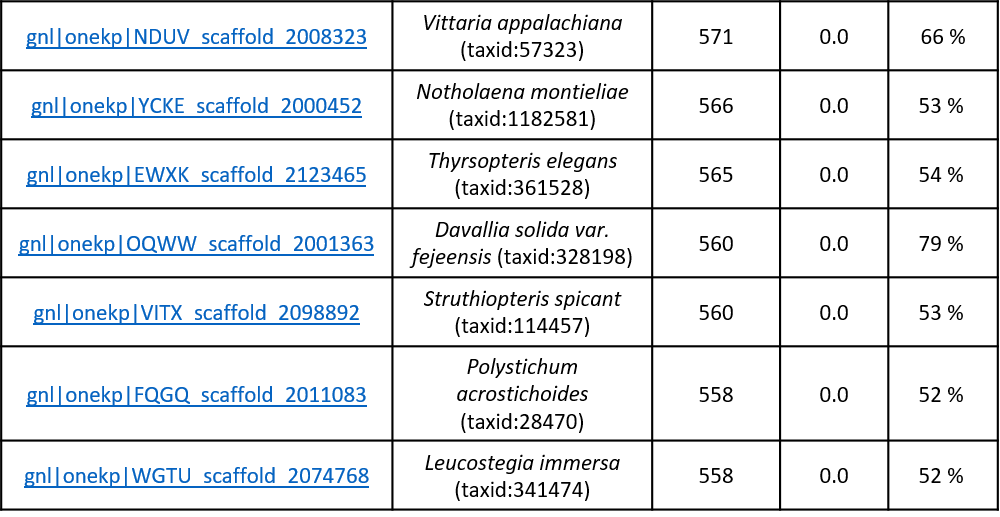
*

BLASTP search of 74 fern transcriptomes from the OneKP database to identify fern species harboring FOS1 encoding gene sequences with the highest sequence identity to *PaqFOS1.*

Supplementary Table 4.

| Analyte | Retention time  [min] | Q1  [*m/z*] | Q3  [*m/z*] | Collision energy  [eV] |
| --- | --- | --- | --- | --- |
| Pipecolic acid [M+H]^+^ | 0.93 | 130.1 | 84.2 ^Q^ | 12 |
|  |  | 130.1 | 56.2 | 25 |
| *N*-hydroxy pipecolic acid [M+H]^+^ | 1.37 | 146.0 | 100.1 ^Q^ | 8 |
|  |  | 146.0 | 70.2 | 15 |
|  |  | 146.0 | 110.1 | 9 |
| Phenylalanine [M+H]^+^ | 2.25 | 166.2 | 120.2 ^Q^ | 14 |
| *N*-hydroxyphenylalanine [M+H]^+^ | 2.53 | 182.1 | 136.0 ^Q^ | 12 |
|  |  | 182.1 | 117.0 | 22 |
| (*E*)-phenylacetaldoxime [M+H]^+^ | 3.78 | 136.1 | 118.1 ^Q^ | 10 |
|  |  | 136.1 | 58.3 | 12 |
|  |  | 136.1 | 91.1 | 30 |
| (*Z*)-phenylacetaldoxime [M+H]^+^ | 3.94 | 136.1 | 118.1 ^Q^ | 10 |
|  |  | 136.1 | 58.3 | 12 |
|  |  | 136.1 | 91.1 | 30 |

Multiple reaction monitoring (MRM) transitions for LC-MS/MS analysis and identification of possible substrates and metabolites formed. Q: identifies the quantifier ion, additional transitions were used for identification only
