# Supplementary material for: A flavin-dependent monooxygenase catalyzes the initial step in cyanogenic glycoside synthesis in ferns
Source: Commun Biol. 2020 Sep 11;3:507. doi: 10.1038/s42003-020-01224-5 (PMC7486406; doi:10.1038/s42003-020-01224-5)
Supplement: Supplementary file 2 — Description of Additional Supplementary Items [file 42003_2020_1224_MOESM2_ESM.docx]

Description of **Additional Supplementary datafiles**

**Supplementary Data 1** Fasta file with complete FMO contigs from *de novo* transcriptomes referred to in this study.

Supplementary Data 2 List of sequence IDs employed in building the phylogenetic tree.

Supplementary Data 3 Supplementary datafiles for metabolite detection *in planta* and *in vitro*
