# Supplementary material for: A flavin-dependent monooxygenase catalyzes the initial step in cyanogenic glycoside synthesis in ferns
Source: Commun Biol. 2020 Sep 11;3:507. doi: 10.1038/s42003-020-01224-5 (PMC7486406; doi:10.1038/s42003-020-01224-5)
Supplement: Supplementary file 6 — Reporting Summary [file 42003_2020_1224_MOESM6_ESM.pdf]

nature research

Corresponding author(s): Birger Lindberg Møller, Elizabeth H. J. Neilson

Last updated by author(s): 11/06/2020

Reporting Summary

Nature Research wishes to improve the reproducibility of the work that we publish. This form provides structure for consistency and transparency in reporting. For further information on Nature Research policies, see Authors & Referees and the Editorial Policy Checklist.

Statistics

For all statistical analyses, confirm that the following items are present in the figure legend, table legend, main text, or Methods section.

n/a	Confirmed	
☐	☒	The exact sample size (*n*) for each experimental group/condition, given as a discrete number and unit of measurement
☐	☒	A statement on whether measurements were taken from distinct samples or whether the same sample was measured repeatedly
☒	☐	The statistical test(s) used AND whether they are one- or two-sided<br>*Only common tests should be described solely by name; describe more complex techniques in the Methods section.*
☒	☐	A description of all covariates tested
☒	☐	A description of any assumptions or corrections, such as tests of normality and adjustment for multiple comparisons
☐	☒	A full description of the statistical parameters including central tendency (e.g. means) or other basic estimates (e.g. regression coefficient) AND variation (e.g. standard deviation) or associated estimates of uncertainty (e.g. confidence intervals)
☒	☐	For null hypothesis testing, the test statistic (e.g. *F*, *t*, *r*) with confidence intervals, effect sizes, degrees of freedom and *P* value noted<br>*Give P values as exact values whenever suitable.*
☒	☐	For Bayesian analysis, information on the choice of priors and Markov chain Monte Carlo settings
☒	☐	For hierarchical and complex designs, identification of the appropriate level for tests and full reporting of outcomes
☒	☐	Estimates of effect sizes (e.g. Cohen's *d*, Pearson's *r*), indicating how they were calculated

Our web collection on statistics for biologists contains articles on many of the points above.

Software and code

Policy information about availability of computer code

Data collection

not applicable

Data analysis

Contributer /author indicated in bracket after each:

Traces of total ion current and of extracted ion currents for specific [M+Na]+ and [M+H]+ adduct ions were used to identify the eluted constituents using Compass DataAnalysis software (version 4.2, Bruker Daltonics)(ST,MS,AKB)

Identification of gene families was performed using the OrthoMCL pipeline (http://orthomcl.org/orthomcl/about.do).(Biosequentia)

Differential gene expression analyses based on the P. aureum and P. aquilinum transcriptomes were carried out using eXpress and the data transferred into R and analyzed with the package NOISeq. (Biosequentia)

For TQ analysis : Bruker MS Workstation software (Version 8.2.1, Bruker, Bremen, Germany) was used for data acquisition and processing(CC)

Bioinformatics/phylogeny:
FMO sequences were obtained using BLASTp 2.2.26+ (ST)
Else the software used for alignments and trees , MEGA 7.0 (ST)

For manuscripts utilizing custom algorithms or software that are central to the research but not yet described in published literature, software must be made available to editors/reviewers. We strongly encourage code deposition in a community repository (e.g. GitHub). See the Nature Research guidelines for submitting code & software for further information.

Data

Policy information about availability of data

All manuscripts must include a data availability statement. This statement should provide the following information, where applicable:

- Accession codes, unique identifiers, or web links for publicly available datasets
- A list of figures that have associated raw data
- A description of any restrictions on data availability

For all data used a source is ascribed; Either as open source dataset with link and description or as supplemental material (Data S1, S2)

Field-specific reporting

Please select the one below that is the best fit for your research. If you are not sure, read the appropriate sections before making your selection.

☒ Life sciences ☐ Behavioural & social sciences ☐ Ecological, evolutionary & environmental sciences

For a reference copy of the document with all sections, see nature.com/documents/nr-reporting-summary-flat.pdf

Life sciences study design

All studies must disclose on these points even when the disclosure is negative.

Sample size	The fern species were chosen based on published literature reports of cyanogenic glyucoside content, and confirmed by our own hands by LC-MS analysis. RNA was extracted from identified tissue at least twice, with the highest quality RNA used for downstream transcriptomic analysis.
Data exclusions	no data exclusions
Replication	Functional characterization by agroinfiltration in Nicotiana benthamiana plants was repeated in three independent experiments, using two biological N. benthamiana replicates, and three technical replicates each time. Similarly, in vitro enzyme assays in E. coli were repeated in three independent experiments with three technical replicates each time
Randomization	Samples for LC-MS was not randomized, howewer standards were run every 10 sample
Blinding	not applicable

Reporting for specific materials, systems and methods

We require information from authors about some types of materials, experimental systems and methods used in many studies. Here, indicate whether each material, system or method listed is relevant to your study. If you are not sure if a list item applies to your research, read the appropriate section before selecting a response.

Materials & experimental systems

n/a	Involved in the study	
☒	☐	Antibodies
☒	☐	Eukaryotic cell lines
☒	☐	Palaeontology
☒	☐	Animals and other organisms
☒	☐	Human research participants
☒	☐	Clinical data

Methods

n/a	Involved in the study	
☒	☐	ChIP-seq
☒	☐	Flow cytometry
☒	☐	MRI-based neuroimaging
